# Development of lyophilized mRNA-LNPs with high stability and transfection efficiency in specific cells and tissues

**DOI:** 10.1093/rb/rbaf023

**Published:** 2025-04-10

**Authors:** Ting Wang, Tao Yu, Wanqi Li, Jianyang Chen, Sitian Cheng, Zeyu Tian, Tzu-Cheng Sung, Akon Higuchi

**Affiliations:** State Key Laboratory of Ophthalmology, Optometry and Visual Science, Eye Hospital, Wenzhou Medical University, Wenzhou, Zhejiang 325027, China; State Key Laboratory of Ophthalmology, Optometry and Visual Science, Eye Hospital, Wenzhou Medical University, Wenzhou, Zhejiang 325027, China; State Key Laboratory of Ophthalmology, Optometry and Visual Science, Eye Hospital, Wenzhou Medical University, Wenzhou, Zhejiang 325027, China; State Key Laboratory of Ophthalmology, Optometry and Visual Science, Eye Hospital, Wenzhou Medical University, Wenzhou, Zhejiang 325027, China; State Key Laboratory of Ophthalmology, Optometry and Visual Science, Eye Hospital, Wenzhou Medical University, Wenzhou, Zhejiang 325027, China; State Key Laboratory of Ophthalmology, Optometry and Visual Science, Eye Hospital, Wenzhou Medical University, Wenzhou, Zhejiang 325027, China; State Key Laboratory of Ophthalmology, Optometry and Visual Science, Eye Hospital, Wenzhou Medical University, Wenzhou, Zhejiang 325027, China; State Key Laboratory of Ophthalmology, Optometry and Visual Science, Eye Hospital, Wenzhou Medical University, Wenzhou, Zhejiang 325027, China; Department of Chemical and Materials Engineering, National Central University, Jhongli, Taoyuan 320, Taiwan, China; R&D Center for Membrane Technology, Chung Yuan Christian University, Chungli, Taoyuan 320, Taiwan, China

**Keywords:** lipid nanoparticles, I-optimal design of mixture experiments, lyophilization, stability, transfection efficiency

## Abstract

Lipid nanoparticles (LNPs) are critical for the delivery of drugs and nucleic acids. However, current mRNA-LNP formulations require stringent freezing for storage, which limits their global distribution. Our previous studies demonstrated that optimizing the lipid type or molar ratio of Comirnaty-type mRNA-LNPs could enhance their lyophilization stability, thus improving their long-term storage stability under mild conditions. This study aims to enhance the storage stability of Spikevax-type mRNA-LNPs by optimizing lipid compositions and utilizing lyophilization for storage at 4°C. Fifteen mRNA-LNP formulations were evaluated for their physicochemical properties and transfection efficiency (TE) in human embryonic kidney (HEK)-293T cells using the I-optimal design of mixture experiments. Mathematical models were developed to predict the relationships among encapsulation efficiency, transfection performance and lipid ratios. The optimized mRNA-LNP formulation (N4), with a 1,2-distearoyl-sn-glycero-3-phosphocholine (DSPC)-to-cholesterol ratio of 0.36, exhibited superior stability and TE after lyophilization. N4 outperformed the original Spikevax formulation in several cell lines, including eye-derived ARPE-19 cells and lung-derived A549 cells. *In vivo*, N4 demonstrated high TE in the spleen of C57BL/6 mice both before and after lyophilization, with no signals observed in the kidneys, heart or eyes. These findings suggest that the optimized N4 formulation offers a robust, stable and efficient delivery system for gene therapy and vaccines, potentially overcoming the storage limitations of current Spikevax-type mRNA-LNPs and broadening their therapeutic applications.

## Graphical abstract

**Figure:**
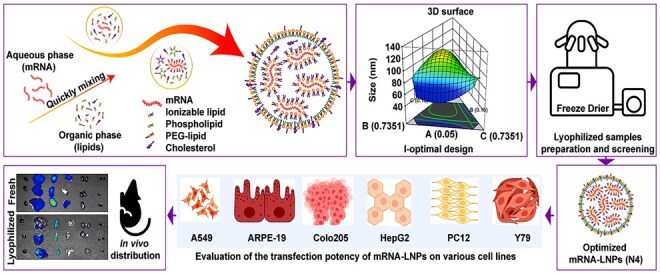

## Introduction

The development of lipid nanoparticles (LNPs) as novel drug delivery systems has significantly advanced gene therapy [1–5]. LNPs offer high biocompatibility, efficient cellular uptake and low immunogenicity, making them ideal nonviral carriers for nucleic acid drugs such as DNA, siRNA and mRNA [6–9]. These properties enable LNPs to encapsulate nucleic acid drugs, protect them from degradation in the serum and facilitate their entry into host cells to exert their therapeutic effects [10–13].

The utility of LNPs was clearly demonstrated during the global SARS-CoV-2 pandemic [14–16]. mRNA vaccines such as Comirnaty^@^ and Spikevax^@^, which are delivered via LNPs, have become crucial tools for mitigating viral transmission [16]. However, a significant challenge with current mRNA-LNP formulations is their requirement for storage at low temperatures (−15°C to −50°C) [17]. These stringent conditions increase costs and complicate distribution, particularly in developing countries that lack robust cold chain logistics [1, 18, 19].

Recent progress in lyophilization, or freeze-drying, techniques for mRNA-LNPs has shown promise in addressing these challenges [20–24]. Lyophilization can significantly enhance the stability of mRNA-LNPs, allowing them to be stored at more manageable temperatures, such as 4°C [21]. Studies by Muramatsu *et al.*, Kafetzis *et al.*, and others have demonstrated that lyophilized mRNA-LNPs maintain their structural integrity, encapsulation efficiency, and transfection performance over extended storage periods [20, 21, 25]. This advancement is crucial for making mRNA-based therapies and vaccines more accessible globally, as it reduces dependency on cold chain logistics and lowers distribution costs [26].

Kafetzis *et al.* [20] investigated the impact of storage temperature on LNPs consisting of CL4H6 (a pH-sensitive cationic lipid), 1,2-dioleoyl-sn-glycero-3-phosphoethanolamine (DOPE) and 1,2-dimyristoyl-rac-glycero-3-methoxypolyethylene glycol-2000 (DMG-PEG_2000_) at a molar ratio of 50:50:1. They demonstrated that cryoprotectants effectively protect these nanoparticles against loss of function and degradation across all storage conditions (RT, 4°C, −80°C) [20]. Muramatsu *et al.* [21] demonstrated that nucleoside-modified mRNA-LNPs containing (6Z, 16Z)-12-((Z)-dec-4-en-1-yl)docosa-6,16-dien-11-yl 5-(dimethylamino)pentanoate (ionizable lipid), 1,2-distearoyl-sn-glycero-3-phosphocholine (DSPC), cholesterol (Cho) and PEG_2000_-C-DMA at a molar ratio of 50:10:38.5:1.5 can be successfully lyophilized. Kim *et al.* [27] investigated the optimal storage conditions for mRNA-LNP formulations consisting of TT3, Dlin-MC3-DMA, DOPE, cholesterol and DMG-PEG_2000_ in ethanol at a molar ratio of 10:25:20:40:5. Previously, our studies demonstrated that optimizing the lipid type or molar ratio of Comirnaty-type mRNA-LNPs can enhance their lyophilization stability [28]. These advances indicated that the lipid composition and molar ratios of mRNA-LNPs not only play pivotal roles in determining their transfection efficiency (TE) but also can influence their stability during lyophilization [1, 29–32]. However, much remains to be learned about the mRNA-LNP compositions used in FDA-approved Spikevax^@^ vaccines (S type), in which heptadecan-9-yl 8-((2-hydroxyethyl)(6-oxo-6-(undecyloxy)hexyl)amino)octanoate (SM-102, ionizable lipid), DSPC (phospholipid), cholesterol and DMG-PEG_2000_ (polyethylene glycol lipid) are included at a molar ratio of 50:10:38.5:1.5.

This study focused on improving the storage stability of mRNA-LNPs (S type) by preparing lyophilized formulations and optimizing the lipid composition. We screened mRNA-LNPs with superior physicochemical properties (diameter and encapsulation efficiency) and TE in human embryonic kidney (HEK)-293T cells by using the I-optimal design of mixture experiments. We established mathematical models to improve our understanding of the relationships among LNP diameter, encapsulation efficiency, normalized median fluorescence intensity (nMFI) [33] and the molar ratios of the four lipids in the mRNA-LNPs. The long-term storage stability of these mRNA-LNPs, which were designed through mathematical modeling [34], was evaluated by examining their physicochemical properties and TE in HEK-293T cells after 0, 1, 4, 8, and 12 weeks. Several cell lines derived from different organs (the lung, liver, eyes, and colon) were selected to systematically evaluate the TE of the optimized mRNA-LNP, named N4. Additionally, we evaluated the *in vivo* distribution of N4 in the major organs of C57BL/6 mice. The potent stability and transfection ability of N4 in both *in vitro* and *in vivo* settings suggest its potential for clinical applications.

## Materials and methods

### Materials

SM-102 was purchased from Shochem Co., Ltd (Shanghai, China), and DMG-PEG_2000_, DSPC and cholesterol were purchased from Sigma–Aldrich Trading Co. Ltd (Shanghai, China). mRNAs encoding enhanced green fluorescence protein (eGFP, CAG EGFP mRNA (N′-Me-Pseudo UTP, 1 mg/ml), cat. # DD4503-02) or firefly luciferase (*FLuc* mRNA (N1-Me-Pseudo UTP, 1 mg/ml), cat. # DD4511-02) were purchased from Vazyme Biotech Co., Ltd (Nanjing, China). Bright-Glo luciferase assay substrate was obtained from Promega (Madison, WI). The Quant-it RiboGreen Assay kits (cat. # R11490) were purchased from Thermo Fisher Scientific (Waltham, MA, USA). HEK-293T, human lung adenocarcinoma cells (A549), retinoblastoma cells (Y79), human colon carcinoma cells (Colo205), human hepatocellular carcinoma cells (HepG2) and rat pheochromocytoma cells (PC12) were purchased from iCell Bioscience, Inc. (Shanghai, China), and each cell line was recently authenticated and tested for mycoplasma contamination. ARPE-19 cells were donated by Professor Quankui Lin’s group at the Eye Hospital of Wenzhou Medical University. Fetal bovine serum (FBS), Dulbecco’s modified Eagle’s medium (DMEM) and phosphate-buffered saline (PBS) were also obtained from Sigma–Aldrich Trading Co. Ltd (Shanghai, China).

### I-Optimal design of mRNA-LNPs with different molar ratios

We used the I-optimal design of mixture experiments for the design of mRNA-LNPs with different molar ratios of lipids in this study, as this approach minimizes the average prediction variance across the design space [35, 36]. Supplementary Table S1 shows the preset range of the four experimental factors, which were designed according to the original lipid molar ratio used in the FDA-approved Spikevax^@^ vaccine. While a completely randomized full factorial design would lead to 4^4^ = 256 runs, the fractional factorial I-optimal design implemented in Design-Expert software (StatEase, Minneapolis, USA) generated a total of 15 runs. The factor levels used in the 15 experimental runs proposed by the Design Expert software are presented in Table 1.

**Table 1.** Experimental groups in the actual I-optimal design of mixture experiments (%)^a^

mRNA-LNPs	DSPC	SM-102	Cholesterol	DMG-PEG_2000_
M01	19	65	15	1
M02	5	35	50	10
M03	18.3	35.9	35.8	10
M04	5	53	38	4
M05	40	15	35	10
M06	40	29.5	29.5	1
M07	14.9	51.2	26.2	7.7
M08	5	65	29	1
M09	23	15	58	4
M10	8.3	65	16.7	10
M11	40	39.5	15	5.5
M12	5	20	65	10
M13	29.9	40.7	26.2	3.2
M14	5	29	65	1
M15	24.7	26.2	45.9	3.2
**MS**	**10**	**50**	**38.5**	**1.5**

a MS represents mRNA-LNPs mimicking the original lipid ratios of LNPs in the commercial Spikevax^@^ vaccine (produced by Moderna) and was not included in the modeling of the subsequent data from the I-optimal design of mixture experiments. It was solely used for validation purposes. DSPC: 1,2-distearoyl-sn-glycero-3-phosphocholine; SM-102: heptadecan-9-yl 8-((2-hydroxyethyl)(6-oxo-6-(undecyloxy)hexyl)amino)octanoate; DMG-PEG_2000_: 1,2-dimyristoyl-rac-glycero-3-methoxypolyethylene glycol-2000.

### Preparation and characterization of mRNA-LNPs with different molar ratios

*FLuc-* or *eGFP*-labeled mRNA was encapsulated in LNPs via a previously described method [37]. Briefly, an acidic aqueous phase (citrate buffer, 10 mM) containing mRNA (27.4 μg/ml) at pH 4.5 was prepared, and the organic phase was prepared by solubilizing ionizable lipid (SM-102), helper phospholipid (DSPC), cholesterol and lipid-anchored PEG (DMG-PEG_2000_) in ethanol at a specified molar ratio (Table 1). Then, the two phases were mixed together at a volume ratio (aqueous solution:ethanol) of 3:1 by pipetting to generate mRNA-LNPs, and the mRNA and ionizable lipids were combined at N:P ratio of 6:1. The prepared mRNA-LNPs were then lyophilized or stored under predetermined test conditions (4°C).

The particle size, polydispersity index (PDI) value and zeta potential of the mRNA-LNPs were measured using a NanoZS Zetasizer (Malvern, Worcestershire, UK). The fresh samples were tested immediately after preparation. Lyophilized mRNA-LNPs were stored for set times (e.g. 0, 1, 4, 8 and 12 weeks) at 4°C. Lyophilized mRNA-LNPs were reconstituted via the rapid addition of nuclease-free water and gently mixed. Particle size measurements were performed with 10 s run durations and the number of runs was manually determined. The mRNA encapsulation efficiency was determined using the Quant-it RiboGreen assay, as previously reported [21]. For TEM imaging, mRNA-LNP samples were prepared using negative staining with 2% phosphotungstic acid and then imaged using a TEM (JEM-F200, JEOL, Japan or FEI Talos F200S G2, Thermo Fisher Scientific, USA).

### Lyophilization process of the mRNA-LNPs

PBS solutions containing 0%, 4%, 10%, 20%, 24%, 30% or 40% (w/v) cryoprotectant (sucrose) were prepared first. Then, freshly prepared mRNA-LNPs were diluted with PBS solutions containing different concentrations of sucrose (1:1, v/v). The final concentrations of sucrose in the mRNA-LNP solutions were 0%, 2%, 5%, 10%, 12%, 15% or 20% (w/v). The mRNA-LNP solutions were subsequently divided into two parts. One part of each freshly prepared sample was used for experiments immediately. The other part was lyophilized in a glass chamber for 12 h using a lyophilizer (SCIENTZ-10/C, Ningbo Scientz Biotechnology, Ningbo, China). Specifically, mRNA-LNP solutions were frozen at −30°C for 3 h, followed by a primary dry cycle at −25°C under vacuum (5–10 Pa) for 16–18 h. During the secondary dry cycle, the samples were heated to RT (22–27°C) under vacuum (20 Pa) for 5 h. The tubes containing lyophilized mRNA-LNPs were stored at 4°C for 0, 1, 4, 8 and 12 weeks to evaluate the stability of the lyophilized mRNA-LNPs.

### Mathematical modeling based on I-optimal design of mixture experiments

We used the Box–Cox transformation to process the experimental data; this is a widely used technique that converts nonnormally distributed data to an approximately normal distribution [38, 39]. This transformation is essential because many statistical methods and machine learning algorithms, such as linear regression, analysis of variance and various hypothesis tests, rely on the assumption that the data are normally distributed.

During the model variable selection stage, we used the sequential model sum of squares, also known as type I sum of squares, to assess the contribution of each variable [40]. This method is sensitive to the order in which variables are introduced. The variables were sequentially added to the model, as outlined in Supplementary Table S2, and the change in the sum of squares was calculated after each addition. The initial parameters of the model were determined once the model showed no significant improvement.

Finally, the Bayesian information criterion (BIC) was used for model selection and variable screening. The BIC penalizes the likelihood function of the model to balance model complexity and goodness of fit, and two common stepwise selection methods, forward selection and backward elimination, are used for variable selection. In forward selection, the model starts with no variables, and those that improve its predictive ability the most are gradually added. Conversely, backward elimination begins with a model containing all candidate variables, and those that contribute least to its predictive power are gradually removed.

By combining the BIC with these two common stepwise selection methods, the model was effectively simplified, and some statistically insignificant parameters were eliminated, thus establishing a model that was both parsimonious and predictive.

We obtained the initial parameters for three simplified models via forward selection and backward elimination on the experimental data, as shown in Supplementary Table S3. We subsequently fitted the models to the experimental data on the basis of the parameters in Supplementary Table S3.

### Cell culture

*In vitro* transfection of mRNA-LNPs was performed with the HEK-293T, Colo205, PC12, ARPE19, HepG2, Y79 and A549 cell lines. Each cell line was cultured in humidified 5% CO_2_ at 37°C during the experiments. Detailed information on the cell culture medium, centrifugation parameters and digestion time for each cell line used in this study is presented in Supplementary Table S4. Cells at passages 3–30 were used for the experiments.

### Evaluation of the cellular uptake and mRNA expression of the mRNA-loaded LNPs *in vitro*

HEK-293T cells, which are cells that exhibit an epithelial morphology and were originally isolated from human embryonic kidney tissue, were grown in DMEM supplemented with 10% FBS at 37°C in a 5% CO_2_ environment via a standard protocol. The cells were detached from polystyrene (TCP) dishes using 0.25% trypsin with EDTA and washed with DMEM via a standard protocol. Subsequently, HEK-293T cells were seeded in transparent 24-well TCP plates at a density of 2 × 10^5^ cells per well. The cells were cultured overnight and then treated with mRNA (*eGFP*)-loaded LNPs at a concentration of 800 ng of mRNA/well. Cellular uptake efficiency and eGFP expression were determined via fluorescence microscopy (Zeiss Model Axio Observer A1, Carl Zeiss, Germany). The TE and fluorescence intensity were evaluated via flow cytometry (BD Accuri™ C6 Plus, BD Biosciences, USA) after 18–24 h of transfection with the mRNA-LNPs. HEK-293T cells transfected with mRNA using a commercial reagent (Lipofectamine 2000, Thermo Fisher Scientific, Waltham, MA, USA) were used as a positive control. Following this method, we also evaluated the TE of the mRNA-LNPs in several other cell lines (Colo205, PC12, Y79, HepG2, ARPE-19 and A549 cells).

### Cell viability assay

The cell viability of the tested cells (HEK-293T, A549, ARPE-19, Colo205, HepG2, PC12 and Y79) transfected with reconstituted lyophilized mRNA-LNPs of N4 and MS, as well as lipo-mRNA was detected by CCK-8 assays. The tested cells were seeding in 96-well plates and then intervened with the same amount mRNA (215 ng) delivered by LNPs or Lipofectimine 2000 after culturing overnight. 10 µl CCK-8 buffer (A311-02, Vazyme Biotech Co., Nanjing, China) was added into each well and incubated at 37°C for another 1 h. Then the absorbance at 450 nm was measured using a multi-mode microplate reader (Synergy Neo2, Agilent Technologies, USA).

### Evaluation of the organ distribution of mRNA-LNPs *in vivo*

All animal study procedures were approved by the ethics committee of Eye Hospital of Wenzhou Medical University (Permission No: YSG24110705). Each experiment was performed following all relevant and applicable institutional and governmental guidelines and regulations. C57BL/6 mice purchased from Beijing Vital River Laboratory Animal Technology (Beijing, China) were utilized for this animal study. Lyophilized mRNA (*FLuc*)-LNPs stored at 4°C were reconstituted via the addition of UltraPure™ DNase/RNase-Free Distilled Water (10977023, Thermo Fisher Scientific, Waltham, MA, USA) to prepare mRNA-LNP solutions with 0.5 mg/ml mRNA. mRNA-LNPs were diluted with the nuclease-free water and administered via tail vein injection using an insulin syringe (Covidien) with a 50 µl injection volume (*n* = 3). We also administered freshly prepared mRNA-LNP solution (0.2 mg/ml mRNA) to C57BL/6 mice via tail vein injection using an insulin syringe (Covidien) with a 50 µl injection volume (*n* = 3).

Six hours after injection, C57BL/6 mice were intraperitoneally (i.p.) injected with d-luciferin substrate (30 mg/ml) at a dose of 150 mg/kg. At 10–20 min after injection, the mice were euthanized in a CO_2_ chamber with 3% isoflurane (Piramal Health Care Limited). The major organs (the heart, liver, spleen, lung, kidneys and eyes) were subsequently dissected and extracted. The bioluminescence signals of each organ were immediately measured using an *in vivo* imaging system (IVIS^®^, PerkinElmer, Waltham, MA, USA) with Living Image Software v.4.7.4 (PerkinElmer, Waltham, MA, USA). The bioluminescence signals were evaluated via Living Image v.4.5.5 data analysis software (PerkinElmer, Waltham, MA, USA).

### Statistics and reproducibility

All statistical analyses were performed using GraphPad Prism version 10.0.0, GraphPad Software, Boston, Massachusetts USA, www.graphpad.com. An ordinary one-way unpaired ANOVA was performed to compare multiple groups. Quantitative data are shown as mean ± standard error of the mean (SEM) and are considered statistically significant when *P *<* *0.05.

## Results and discussion

### Design and synthesis of mRNA-LNPs (S type) with different lipid molar ratios using the I-optimal design of mixture experiments

**Figure 1.**
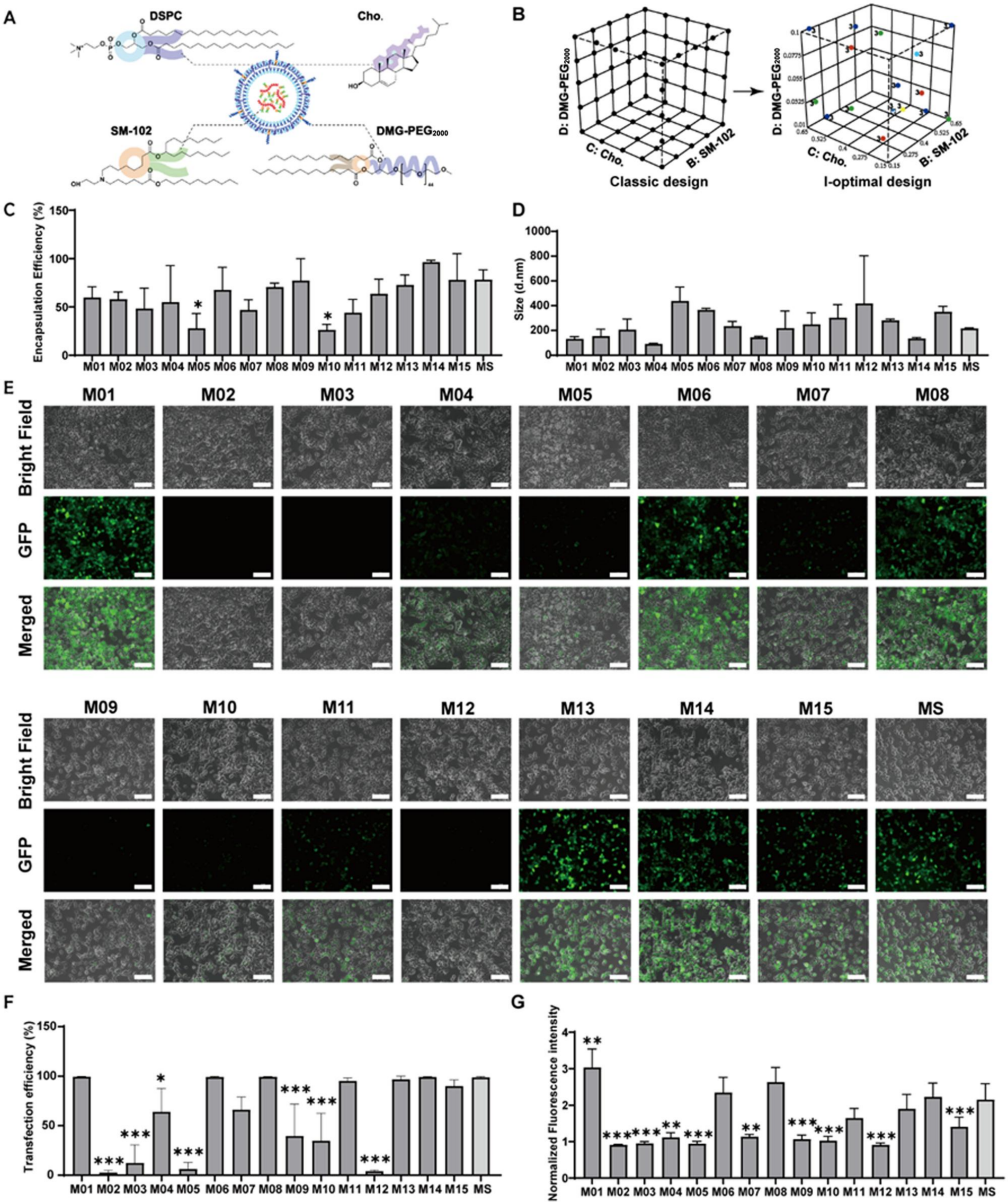
Screening for optimal mRNA (*eGFP*)-LNPs that have high encapsulation and transfection efficiencies via the I-optimal design of mixture experiments. (**A**) Chemical structure of lipids in mRNA-LNP (MS type). (**B**) Illustration of the classical design and I-optimal design of mixture experiments. (**C**) Encapsulation efficiency of each designed mRNA-LNP and MS. (**D**) Particle size of each designed mRNA-LNP and MS. (**E**) Fluorescence images of HEK-293T cells treated with each designed mRNA-LNP and MS. The scale bar indicates 50 μm. (**F and G**) TE (**F**) and eGFP expression intensity (**G**) in HEK-293T cells treated with each designed mRNA-LNP, as evaluated by flow cytometry. The four lipids in the mRNA (*eGFP*)-LNPs were SM-102, DMG-PEG_2000_, DSPC and cholesterol. All the data are presented as the mean ± s.d. (*n* = 3). Statistical significance was analyzed by one-way ANOVA. *P <* *0.05, **P <* *0.01, ***P <* *0.001.

To screen freshly prepared mRNA-LNPs with high encapsulation and transfection efficiencies in treated cells, we first used an optimal experimental design to create mRNA-LNPs (S type, Figure 1A) with varying lipid molar ratios. The I-optimal design of mixture experiments (Figure 1B) is a powerful tool for optimizing experiments involving processes, mixtures or combinations of factors and components; this tool can save considerable experimental labor compared with the classical design [35, 36, 41]. In this study, 15 formulations of mRNA-LNPs were designed and prepared with varying molar ratios of SM-102 (ionizable lipid), DMG-PEG_2000_, DSPC (phospholipid) and cholesterol, and the mRNA encoding eGFP was entrapped in the LNPs. The molar ratio ranges for each type of lipid are presented in Table 1. Specifically, the molar ratios for SM-102 and cholesterol were between 15% and 65%, those for DSPC were between 5% and 40%, and those for PEG lipids were between 1% and 10%. These ranges were based on the original lipid molar ratios in Spikevax-type mRNA-LNPs (referred to as MS).

### Characteristics of mRNA-LNPs and evaluation of the TE of mRNA-LNPs in HEK-293T cells

We evaluated the effects of the lipid molar ratio on the diameter and encapsulation efficiency of the 15 designed mRNA-LNPs, as well as their TE and fluorescence intensity in HEK-293T cells. This allowed us to identify mRNA-LNPs with relatively high encapsulation and transfection efficiencies. mRNA-LNP (MS) was used as a positive control. The results are shown in Figure 1.

The encapsulation efficiency of mRNA in mRNA-LNPs (MS) was ∼78%, as shown in Figure 1C, and the values of 13 of the designed mRNA-LNPs ranged from 44% to 96%, with no significant differences compared with those of MS, except for M05 and M10, which had an encapsulation efficiency of ∼27%. The particle sizes (Figure 1D) of all 15 designed mRNA-LNPs ranged from 93 to 440 nm, with no significant differences compared with those of the mRNA-LNPs (MS), which had a diameter of 217 nm. The PDI values (Supplementary Figure S1) of most designed mRNA-LNPs and MS were below 0.4, except for M10, M12 and M15, indicating a higher degree of inhomogeneity in these specific mRNA-LNP formulations.

The TE of the 15 designed mRNA-LNPs and mRNA-LNPs (MS), as well as the nMFI of eGFP expression in treated HEK-293T cells, are shown in Figure 1E–G and Supplementary Figure S2. The nMFI was calculated by normalizing the MFI of HEK-293T cells treated with mRNA-LNPs to the MFI of the negative control, providing a more accurate description of the cell population in culture. The TE of mRNA-LNPs M01, M06, M08, M11, M13, M14 and M15 in HEK-293T cells was ∼100%, which was comparable to that of mRNA-LNPs (MS), with no significant differences in TE among these samples. The nMFI of mRNA-LNPs M06, M08, M11, M13 and M14 in the HEK-293T cells ranged from 1.6–2.7, with no significant differences compared with those treated with mRNA-LNPs (MS), with an nMFI of ∼2.2. However, the nMFI of the HEK-293T cells treated with mRNA-LNPs M01 was significantly greater than that in the cells treated with mRNA-LNPs (MS). mRNA-LNPs M01 were identified as the best formulation, with the highest TE and nMFI among all the mRNA-LNPs designed via the I-optimal design of mixture experiments in this study.

### Model establishment on the basis of the results of the I-optimal design of mixture experiments

We thoroughly explored the relationships between the lipid molar ratio in mRNA-LNPs and their diameter, encapsulation efficiency and fluorescence intensity in HEK-293T cells. This was accomplished through mathematical modeling of the experimental runs. To more accurately evaluate the transfection-related properties of each mRNA-LNP formulation, we used nMFI data [33] instead of TE because the latter often reaches 100% for some mRNA-LNPs, making it less effective at distinguishing between different formulations.

**Figure 2.**
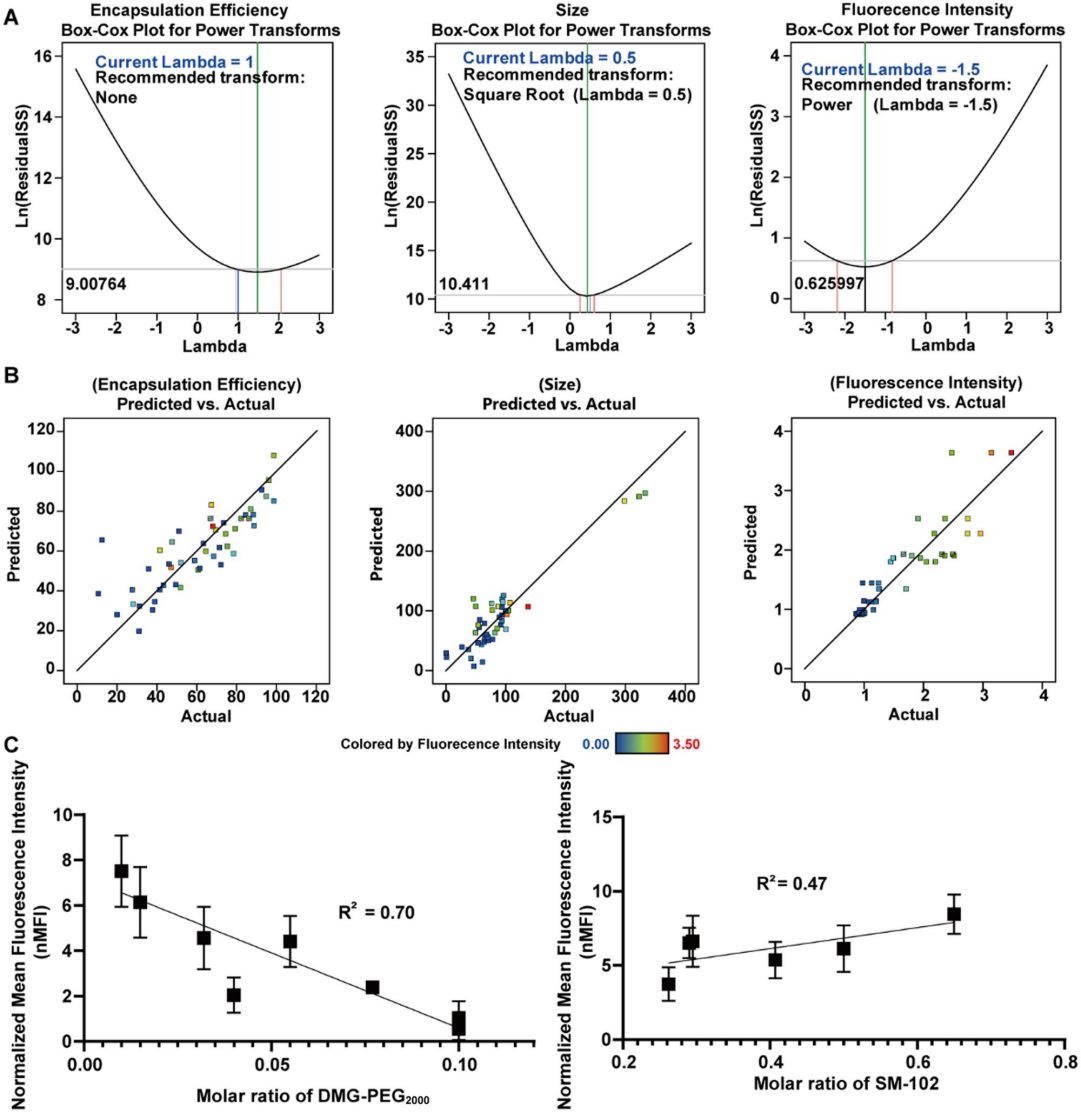
Mathematical modeling based on the results of the I-optimal design of mixture experiments. (**A**) Box–Cox transformation indicator plots for encapsulation efficiency, particle size and fluorescence intensity. The middle vertical lines correspond to the *λ* values for each variable during the normalization process. (**B**) The comparison plot of model-predicted values versus experimental values illustrates that for encapsulation efficiency, particle size and fluorescence intensity; the data points marked ☑ with represent validation data that are not used in model building. All three models show data points closely clustered around the 45° line, further confirming the accuracy of the predictions. (**C**) Illustration of the linear fit of nMFI against the molar ratios of DMG-PEG_2000_ and SM-102 in the I-optimal design of mixture experiments.

Data preprocessing is a crucial initial step in statistical analysis and modeling; this step aims to ensure data quality and enhance the reliability of the results. We first applied a Box–Cox transformation to the dataset to increase the accuracy of the mathematical model (Figure 2A) [38, 39]. This transformation ensures that the data meet the assumption of normality, thereby improving the validity of the subsequent model fit.

In this study, we developed mathematical equations to correlate and identify the influence of formulation variables *A* (phospholipid, DSPC), *B* (ionizable lipid, SM-102), *C* (cholesterol) and *D* (PEG-lipid, DMG-PEG_2000_) on each response variable: encapsulation efficiency, diameter and nMFI. Table 2 and Figure 2B display the regression outcomes for the measured responses. The response values (Table 3) for the encapsulation efficiency (*E*), diameter (*G*) and nMFI (*F*) estimated via the linear model fit well, with *R*^2^ values of 0.6273, 0.8172 and 0.9085, respectively. These relatively high *R*^2^ values, especially for nMFI (*F*), indicate that the statistical models effectively explain the variability in the responses [35]. The discrepancies between the adjusted and predicted *R*^2^ values were 0.08, 0.09 and 0.04, respectively, further demonstrating the reliability of the models, as these differences were <0.2 (Table 3). Additionally, the adequate precision for all three models was >4, which is desirable. Thus, these models can be used to navigate the design space effectively.

**Table 2.** The actual data transformation and fitting results of the three models^a^

Model	Transformation method (*Y*^λ^)	Fitting results
Encapsulation efficiency	*E* = $Y_{1}^{1}$	*E* = 57.77 *A* + 62.05 *B* + 106.35 *C* − 177.89 *D*
Diameter	*G* = $Y_{2}^{0.5}$	*G* = 25.87 *A* + 9.12 *B* + 4.22 *C* − 77.48 *D* − 21.14 *AB* +12.29 *BC* + 49.40 *BD* + 117.57 *CD*
Florescence intensity	*F* = $Y_{3}^{-1.5}$	*F* = − 0.15 *A* + 0.30 *B* + 0.45*C* + 5.61 *D* + 1.67 *AC*

a A: Molar ratio of DSPC in the mRNA-LNPs, B: molar ratio of SM-102 in the mRNA-LNPs, C: molar ratio of cholesterol in the mRNA-LNPs, D: molar ratio of DMG-PEG_2000_ in the mRNA-LNPs. The suitable ranges for each type of lipid are presented in the Supplementary Table S1.

**Table 3.** Statistics of the fitting results of the three models^a^

Model	Std. dev.	Mean	C.V. %	*R*²	Adjusted *R*²	Predicted *R*²	Adequate precision
Encapsulation efficiency	14.31	59.49	24.06	0.6273	0.5987	0.5139	16.84
Diameter	1.56	8.83	17.65	0.8127	0.7686	0.6752	18.27
Florescence intensity	0.0961	0.769	12.5	0.9085	0.8902	0.8491	19.82

a Predicted *R*^2^ and adjusted *R*^2^ are two important indicators of the goodness-of-fit of a linear regression model. If the difference between these two values is <0.2, it generally indicates that the model has reasonable predictive power for new data, as it suggests that the model is not overfitted. Adequate precision measures the ratio of the effective signal to random error (noise) in the model prediction of the response variable. An adequate precision value >4 typically indicates acceptable predictive ability, as it shows that the effective signal is significantly greater than the noise.

Furthermore, the experimental values of encapsulation efficiency, diameter and nMFI obtained for the original Spikevax-type mRNA-LNPs were compared with the values predicted by these models to validate their accuracy. The data are shown in Supplementary Tables S5, S6 and S7. The experimental MS values were close to the predicted values, further demonstrating the predictive ability of the models. In summary, considering the validation data, the three models we developed exhibit strong predictive capabilities within the experimental design space.

We observed a wide range of values in the comparison between the experimental and predicted values in the fluorescence intensity and encapsulation efficiency models, as shown in Figure 2B. This variability raises concerns about data consistency and model accuracy, although the models capture some degree of variation. In contrast, the diameter model showed overly concentrated comparison values of ∼50–100 nm, indicating that it may not fully account for all key factors (such as pH, temperature and stirring rate) that influence particle size. Therefore, only the encapsulation efficiency and nMFI models were used to predict the optimal mRNA-LNP formulations by adjusting the molar ratios of the four lipids in the following experiments.

We found that DMG-PEG_2000_ and the SM-102 (ionizable lipid) significantly influence the final mRNA expression levels through model analysis (Figure 2C). Specifically, the level of PEG lipids was negatively correlated with the mRNA expression level (although a minimum amount of ∼0.5% PEG lipids is reported to be necessary to prevent the aggregation of mRNA-LNPs [42]), whereas the level of ionizable cationic lipids was positively correlated with the mRNA expression level, as shown in Figure 2C. By setting model objectives, we can precisely adjust the composition of LNPs on mRNA-LNPs to achieve optimal encapsulation efficiency and eGFP expression levels. We assigned a higher weight to higher values to ensure high encapsulation efficiency for the final candidates. Additionally, we placed greater importance on the fluorescence intensity model to ensure that the optimized formulation achieves both high TE and high encapsulation efficiency. The specific steps and parameter settings used to optimize the lipid molar ratio are presented in Supplementary Table S8, and the candidate formulations are presented in Supplementary Table S9. The candidate (C1) with the highest predicted nMFI was selected for subsequent experiments.

### Effects of sucrose concentration on the physiochemical properties and TE of the mRNA-LNPs before and after lyophilization

**Figure 3.**
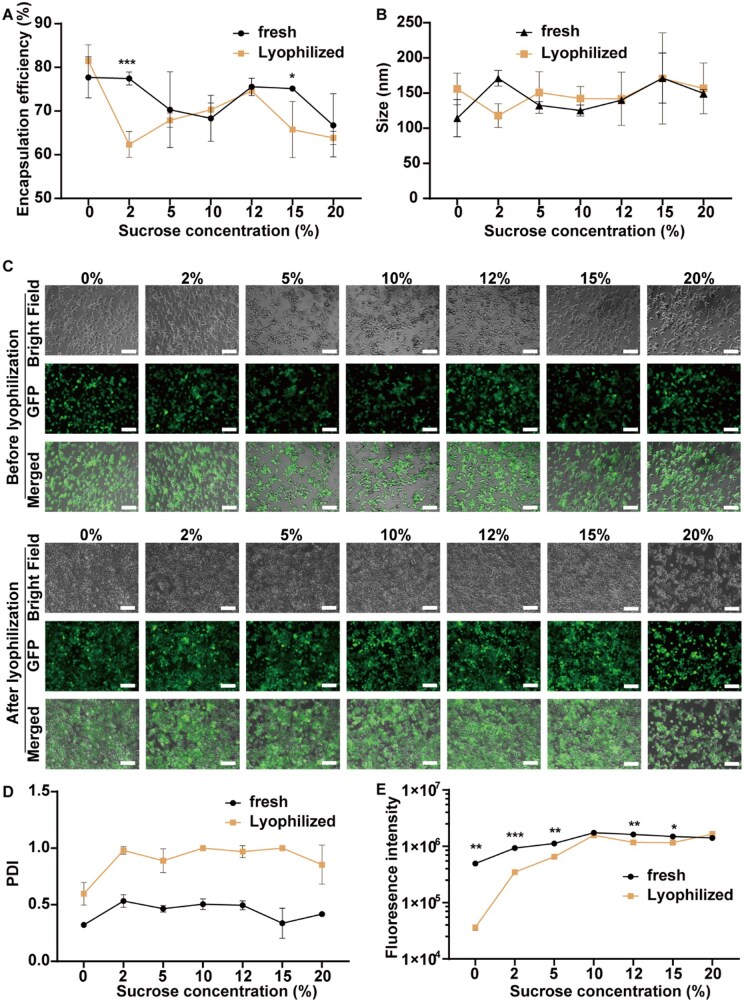
Evaluation of the effects of sucrose concentration on the diameter and encapsulation efficiency of mRNA (*eGFP*)-LNPs and the TE in HEK-293T cells before and after lyophilization. (**A**) Encapsulation efficiency of mRNA in fresh mRNA-LNP mixture with 0%, 2%, 5%, 10%, 12%, 15% and 20% sucrose and lyophilized mRNA-LNPs after reconstitution. (**B and D**) The particle size (**B**) and PDI (**D**) of fresh mRNA-LNP mixture with 0%, 2%, 5%, 10%, 12%, 15% and 20% sucrose and lyophilized mRNA-LNPs after reconstitution. (**C and E**) TE evaluated by fluorescence imaging (**C**) and eGFP expression intensity evaluated by flow cytometry (**E**) in HEK-293T cells treated with fresh mRNA-LNPs with 0%, 2%, 5%, 10%, 12%, 15% and 20% sucrose and lyophilized mRNA-LNPs after reconstitution. The scale bar represents 50 μm. The four lipids in the mRNA (*eGFP*)-LNPs were SM-102, DMG-PEG_2000_, DSPC and cholesterol. The molar ratio of the four components (SM-102:DMG-PEG_2000_:DSPC:cholesterol) was 65:1:19:15. All the data are presented as the mean ± s.d. (*n* = 3). Statistical significance was analyzed by one-way ANOVA. *P <* *0.05, **P <* *0.01, ***P <* *0.001.

Sucrose is one of the most commonly used cryoprotectants in the nanomaterial freezing process [43, 44]. To determine the optimal sucrose concentration for protecting mRNA-LNPs during lyophilization, we prepared M01 mRNA-LNPs with various sucrose concentrations (0%, 2%, 5%, 10%, 12%, 15% and 20%). We evaluated the physicochemical properties (diameter and encapsulation efficiency) of each mRNA-LNP as well as the TE and fluorescence intensity in treated HEK-293T cells. The results are shown in Figure 3A–E.

The average encapsulation efficiency of mRNA in mRNA-LNPs with 2% sucrose significantly decreased from 77% to 62% after lyophilization (*P *<* *0.001), and that in mRNA-LNPs with 15% sucrose significantly decreased from 75% to 66% after lyophilization (*P *<* *0.05), as shown in Figure 3A. The average encapsulation efficiency of mRNA in mRNA-LNPs with 0%, 5%, 10%, 12% and 20% sucrose did not significantly change before and after lyophilization. The diameter (Figure 3B) of the mRNA-LNPs with 2% sucrose decreased from 171 nm to 118 nm after lyophilization, whereas the diameter of those with other sucrose concentrations ranging from 110 nm to 170 nm did not significantly change before and after lyophilization. The PDI values of the mRNA-LNPs with set sucrose concentrations all increased after lyophilization, with increases ranging from 0.3 to 0.7 (Figure 3D).

To further evaluate the structural integrity of mRNA-LNP nanoparticles before and after lyophilization, TEM imaging was performed on representative formulations. Freshly prepared and reconstituted lyophilized mRNA-LNP formulations containing 0%, 5%, 8% and 20% sucrose were analyzed, and the results are presented in Supplementary Figure S3. The images indicate that mRNA-LNP formulations with ∼8% sucrose maintained the best morphology after lyophilization.

The TE of the tested mRNA-LNPs with set sucrose concentrations in HEK-293T cells approached 100% before and after lyophilization, and eGFP expression was observable by fluorescence microscopy in all treated HEK-293T cells (Figure 3C). However, the fluorescence (eGFP) intensities in the HEK-293T cells treated with the mRNA-LNPs containing 0%, 2%, 5%, 12% or 15% sucrose decreased after lyophilization, with the maximum decrease (up to 10 times) observed in the mRNA-LNPs supplemented with 0% sucrose (Figure 3E and Supplementary Figure S4).

Therefore, the optimal sucrose concentration for maintaining the characteristics and bioactivities of mRNA-LNPs after lyophilization is between 5% and 10% on the basis of the results obtained for encapsulation efficiency, particle size and fluorescence intensity. On the basis of the reported literature data [20, 45] and our experimental results, we decided to use an 8% sucrose concentration for further experiments. It should be noted that while ∼8% sucrose was identified as the optimal concentration for lyophilization in Spikevax-type mRNA-LNPs and selected for further experiments, the interaction between nanoparticles and cryoprotectants may vary depending on the specific ionizable lipid used, as summarized in the review article [41]. However, the cryoprotectant concentration could be slightly adjusted and optimized around 8% sucrose, as this concentration generally preserves the structural integrity of mRNA-LNPs under lyophilization conditions for most ionizable lipids, even though it may not always be the absolute optimal choice.

### Evaluation of the long-term storage stability of mRNA-LNPs with various ratios of DSPC to cholesterol

We further evaluated the long-term storage stability of lyophilized mRNA-LNPs after determining the optimal sucrose concentration for maintaining the physicochemical properties and bioactivities of mRNA-LNPs during lyophilization. The results described above suggest that the molar ratios of ionizable lipid and PEG lipid notably affect TE and eGFP expression intensity in HEK-293T cells following treatment with freshly prepared mRNA-LNPs. The optimal molar ratios of SM-102 and DMG-PEG_2000_ were 0.65 and 0.01, respectively, to maintain high TE and eGFP expression intensity.

**Figure 4.**
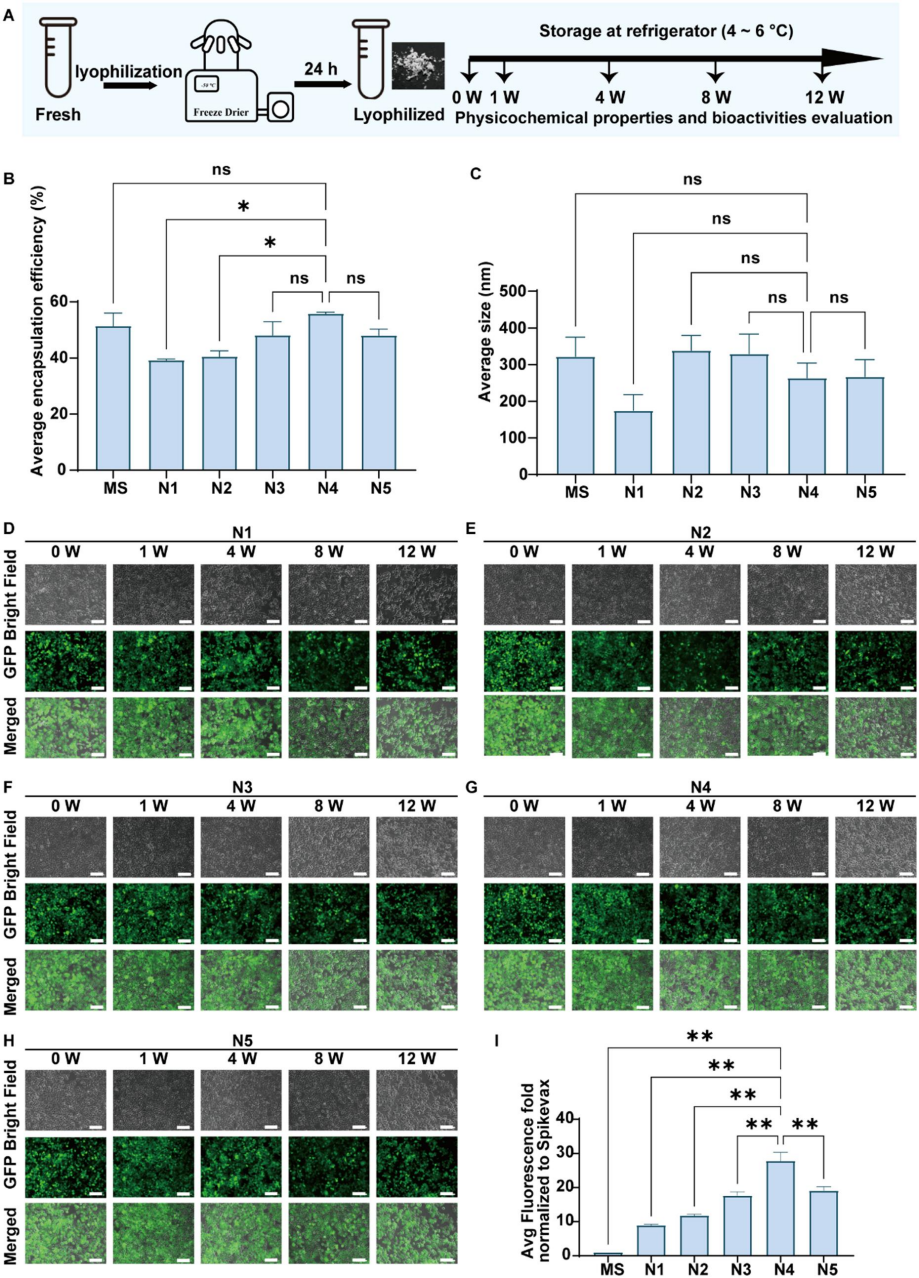
Evaluation of the influence of the DSPC-to-cholesterol ratios (DSPC/Cho) on the long-term storage stability of mRNA-LNPs at 4°C after lyophilization. (**A**) Schematic diagram illustrating the lyophilization process and long-term storage stability evaluation. (**B**) The average encapsulation efficiency of reconstituted lyophilized mRNA-LNPs with various DSPC/Cho ratios (5.8, 2.4, 1.27, 0.36 and 0.13). (**C**) The average particle size of reconstituted lyophilized mRNA-LNPs with various DSPC/Cho ratios (5.8, 2.4, 1.27, 0.36 and 0.13). (**D–H**) Transfection efficiency and eGFP expression intensity in HEK-293T cells treated with reconstituted lyophilized mRNA-LNPs with different DSPC/Cho ratios [5.8 (**D**), 2.4 (**E**), 1.27 (**F**), 0.36 (**G**) and 0.13 (**H**)] after storage for 0, 1, 4, 8 and 12 weeks, as evaluated by fluorescence microscopy. The scale bar represents 50 μm. (I) The average eGFP expression intensity, normalized to MS, in HEK-293T cells treated with reconstituted lyophilized mRNA-LNPs with varying DSPC/Cho ratios, as evaluated by flow cytometry. The four lipids in the mRNA (*eGFP*)-LNPs were SM-102, DMG-PEG_2000_, DSPC and cholesterol. All the data are presented as the mean ± s.d. (*n* = 3). Statistical significance was analyzed by one-way ANOVA. *P <* *0.001; **P <* *0.0001; ns, no significant difference.

Thus, we fixed the molar ratios of SM-102 and DMG-PEG_2000_ at these optimal values and adjusted the molar ratios of DSPC and cholesterol. Five mRNA-LNP formulations (named N1 (C1), N2, N3, N4 and N5) were designed (Supplementary Table S10), with DSPC-to-cholesterol ratios of 5.8, 2.4, 1.27, 0.36 and 0.13, respectively. We evaluated the physicochemical properties (i.e. diameter and encapsulation efficiency) of these designed mRNA-LNPs, as well as their TE and eGFP expression in HEK-293T cells after storage at 4°C for 0, 1, 4, 8 and 12 weeks after lyophilization (Figure 4A).

The lyophilized mRNA-LNPs of N4, which were stored for 0, 1, 4, 8 or 12 weeks, presented the highest average encapsulation efficiency of 58%, as shown in Figure 4B. The average encapsulation efficiency of lyophilized N4 mRNA-LNPs stored for 0, 1, 4, 8 and 12 weeks ranged from 46% to 78%. As shown in Figure 4C, the average size of lyophilized mRNA-LNPs stored for 0, 1, 4, 8 and 12 weeks ranged from ∼185 nm to 320 nm, indicating that there was no significant difference among the tested lyophilized mRNA-LNPs with various molar ratios of DSPC to cholesterol.

The average PDI values (Supplementary Figure S5) of the lyophilized mRNA-LNPs of N1, N2 and N3 were 0.8, 1.0 and 0.7, respectively, which were significantly greater than those of N4 and N5, whose PDI values were 0.5 and 0.4, respectively. The average zeta potential (Supplementary Figure S6 and Supplementary Table S11) of original mRNA-LNP (MS), optimized mRNA-LNP (N1) and mRNA-LNP (N4) remained positive, ranging between 4.3 mV and 9.6 mV both before and after lyophilization. The average zeta potential of freshly prepared Lipofectamine 2000-encapsulated (*eGFP*) mRNA was −12.5 mV, which slightly shifted to −17.4 mV after lyophilization.

The TE of all tested lyophilized mRNA-LNPs, which were stored for 0, 1, 4, 8 or 12 weeks, was ∼100% in this study (Figure 4D–H, and Supplementary Figure S7). As expected, the average fluorescence intensity of all the designed mRNA-LNPs (N1, N2, N3, N4 and N5) was significantly greater than that of mRNA-LNPs (MS) after lyophilization (Figure 4I). Among all the mRNA-LNPs designed in this study, the lyophilized mRNA-LNPs (N4), which were stored for 0, 1, 4, 8 or 12 weeks, presented the highest average eGFP expression intensity, with an average nMFI of 28.

In addition to these biological performance indicators (TE and nMFI), physical properties such as particle size and PDI are also important indicators for evaluating formulation quality. The lyophilized mRNA-LNPs of N4 also showed better performance in terms of these physical parameters, with smaller particle sizes and lower PDI values indicating higher uniformity and stability of the formulation, as shown in Figure 4 and Supplementary Figure 5. In drug delivery systems, a smaller and more uniform particle size helps improve the bioavailability of the formulation and reduce potential toxicity [11, 46].

Stability is a key factor in determining the storage life and transport conditions of lyophilized samples. The lower particle size and PDI value of the mRNA-LNPs (N4), combined with its excellent encapsulation efficiency and fluorescence expression characteristics, imply greater stability during lyophilization, ensuring its efficacy during long-term storage and transport.

The mRNA-LNPs (N4) exhibited superior performance in a comprehensive analysis and comparison with other formulations, including the MS formula, in terms of multiple key performance indicators, demonstrating its potential advantages as a drug carrier. We further validated the efficacy of the mRNA-LNPs (N4) in various cell lines from different tissue sources in subsequent experiments.

### Comparison of the TE of the optimized formulation in cell lines from different tissue sources

**Figure 5.**
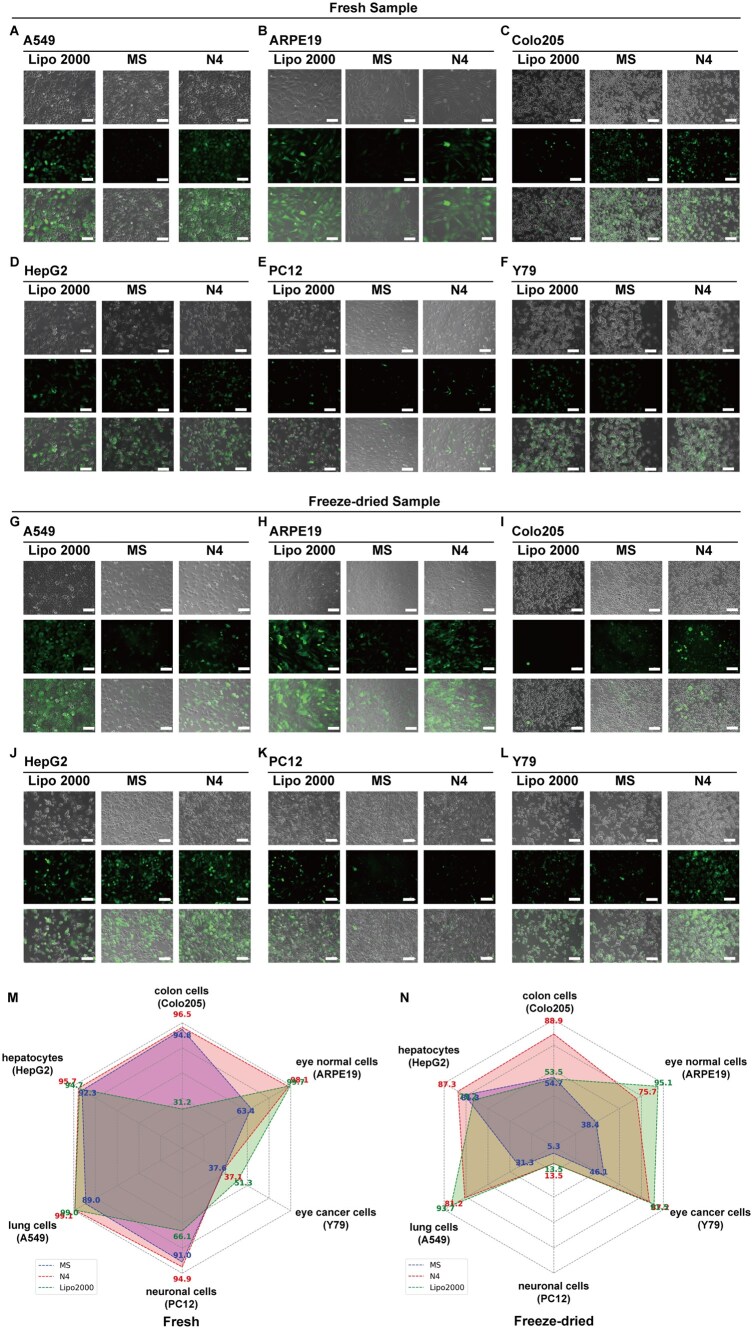
Comparisons of TE for optimized mRNA-LNP (N4), original mRNA-LNP (MS) and Lipofectamine 2000-encapsulated (*eGFP*) mRNAs in various cell lines derived from different organs. (**A–L**) Cell morphology and eGFP expression of each cell type [human lung adenocarcinoma cells (**A** and **G**; A549), human retinal pigment epithelium cells (**B** and **H**; ARPE19), human colon cancer cells (**C** and **I**; Colo205), human hepatocellular carcinoma cells (**D** and **J**; HepG2), cells derived from pheochromocytoma of the rat adrenal medulla (neuronal) (**E** and **K**; PC12) and human retinoblastoma cells (**F** and **L**; Y79)], which were transfected with fresh mRNA-LNPs of N4 and MS as well as Lipofectamine 2000 (**A–F**) and lyophilized mRNA-LNPs of N4 and MS formula as well as Lipofectamine 2000 (**G**–**L**). The scale bar represents 50 μm. (**M** and **N**) Statistical analysis of the TE of fresh (**M**) or lyophilized (**N**) mRNA-LNP (N4), original mRNA-LNP (MS) and Lipofectamine 2000 (Lipo2000) encapsulated (*eGFP*) mRNA in various cell lines derived from different organs, as evaluated by flow cytometry. The four lipids in the mRNA (*eGFP*)-LNPs were SM-102, DMG-PEG_2000_, DSPC and cholesterol.

The HEK-293T cell line was used to screen mRNA-LNP formulations for high TE, eGFP expression intensity and long-term storage stability, as described in the previous sections. This screening led to the identification of the optimal mRNA-LNP formulation, N4. We further aimed to determine whether N4 exhibited superior TE compared with the original Spikevax-type mRNA-LNP (MS) as well as Lipofectamine 2000 (a conventional transfection reagent) across various cell lines derived from different organs (i.e. the lung, liver, eyes and colon). The selected cell lines included human lung adenocarcinoma cells (A549), retinal pigment epithelial cells (ARPE-19), retinoblastoma cells (Y79), human colon carcinoma cells (Colo205), human hepatocellular carcinoma cells (HepG2) and rat pheochromocytoma cells (PC12) which are commonly used as a neuronal cell model [47–49]. We evaluated the TE and eGFP expression of N4 and MS in these cell lines. Additionally, mRNA encapsulated with commercial Lipofectamine 2000 (mRNA-Lipo) served as a positive control to ensure the accuracy and reliability of the results, as shown in Figure 5.

Figure 5A–L shows eGFP expression in each cell type [lung cells (A549), RPE cells (ARPE19), colon cells (Colo205), hepatocytes (HepG2), neuronal cells (PC12) and retinal cells (Y79)], which were transfected with fresh mRNA-LNPs of N4 and MS as well as mRNA-Lipo (Figure 5A–F) and lyophilized mRNA-LNPs of N4 and MS as well as mRNA-Lipo (Figure 5G–L). The TE of fresh and lyophilized mRNA-LNPs of N4 and MS, as well as mRNA-Lipo in each tested cell, is shown in Figure 5M and N, and Supplementary Figure S8.

There was no significant difference in TE (∼100%) in the HepG2 (hepatocyte) and A549 (lung) cell lines between freshly prepared N4, MS and mRNA-Lipo before lyophilization (Figure 5M and N). However, reconstituted lyophilized N4 showed a significantly greater TE (87.3%) than mRNA-Lipo did, which had a TE of 73.2% in HepG2 cells after lyophilization (Figure 5N). In contrast, there was no significant difference in TE between reconstituted lyophilized MS and mRNA-Lipo in HepG2 cells after lyophilization. In A549 cells, reconstituted lyophilized mRNA-Lipo exhibited significantly greater TE (93.7%) than both N4 and MS, which had transfection efficiencies of 81.2% and 31.3%, respectively, after lyophilization. However, the TE of N4 was still significantly greater than that of MS.

In the Colo205 (colon) and PC12 (neuronal) cell lines, N4 exhibited superior TE compared to MS and the mRNA-Lipo both before and after lyophilization (Figure 5M and N). In the Y79 (retinal) cell line, mRNA-Lipo showed superior TE compared to both MS and N4, regardless of whether fresh or lyophilized samples were used.

In ARPE-19 (retinal pigment epithelium) cells, the TE of freshly prepared N4 and mRNA-Lipo was ∼100%, which was significantly greater than the 63.4% efficiency of freshly prepared MS. After lyophilization, the reconstituted mRNA-Lipo had an ∼100% TE, which was significantly greater than that of the reconstituted MS and N4, which had efficiencies of 38.4% and 75.7%, respectively.

The cell viability results (Supplementary Figure S9) demonstrate that the mRNA-LNP formulations of N4 and MS did not significantly affect the viability of HEK-293T, ARPE-19, Colo205, HepG2 and PC12 cells, both before and after lyophilization (reconstituted). In contrast, mRNA-Lipo significantly reduced the viability of Colo205 cells under both conditions. Notably, freshly prepared mRNA-LNP formulations of N4 and MS, as well as mRNA-Lipo, decreased the viability of A549 cells. Additionally, reconstituted lyophilized mRNA-LNP formulations of N4 and MS, along with mRNA-Lipo, reduced the viability of Y79 cells. Overall, N4 exhibited lower cytotoxicity compared to MS and Lipo, suggesting that it may be a more biocompatible mRNA delivery vehicle.

These results indicate that, compared with the original Spikevax-type MS and commercial mRNA-Lipo, the optimized N4 formulation has superior transfection potential across multiple cell lines. These findings provide crucial information for the future design of mRNA delivery systems and will guide further optimization of mRNA-LNPs. Subsequent studies are needed to evaluate their potential and safety after transfection into specific tissues for *in vivo* applications.

### *In vivo* distribution of the optimized mRNA-LNP formulation which was transfected into mice intravenously

**Figure 6.**
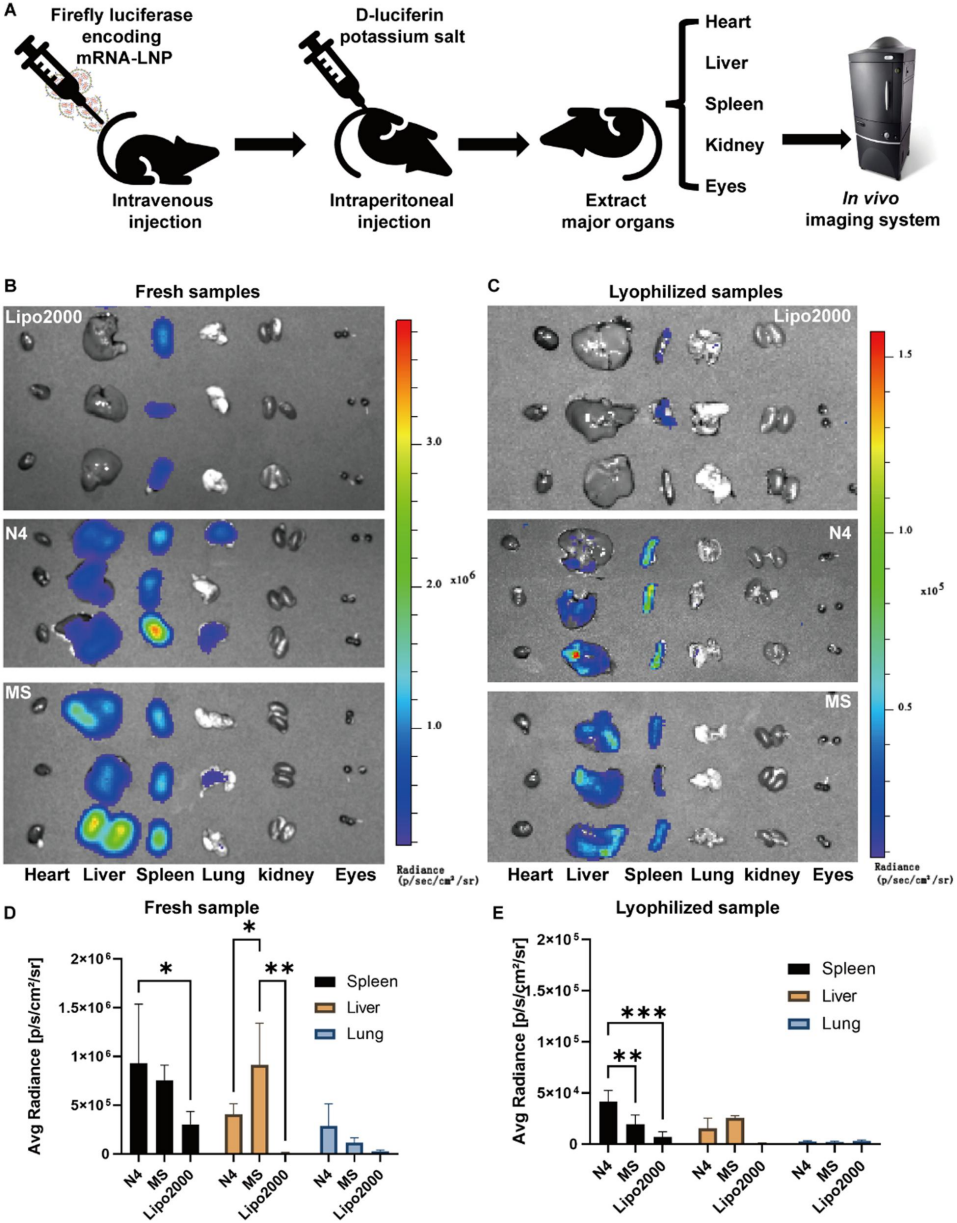
Bioluminescence signals in the major organs (**A**) were detected after tail vein injection of fresh (**B** and **D**) and reconstituted lyophilized (**C** and **E**) optimal mRNA (*FLuc*)-LNPs (N4), original mRNA (*FLuc*)-LNPs (MS) and Lipofectamine 2000-encapsulated (*FLuc*) mRNA. The mice were intravenously injected with 10 µg of (*FLuc*) mRNA using mRNA-LNPs (N4 and MS) and Lipofectamine 2000. Representative IVIS images were captured 6 h after intravenous injection of mRNA *(FLuc)*-LNPs. The sucrose concentration in freshly prepared mRNA *(FLuc)*-LNPs and reconstituted lyophilized mRNA (*FLuc*)-LNPs was fixed at 8%. The four lipids in the mRNA (*FLuc*)-LNPs were SM-102, DMG-PEG_2000_, DSPC and cholesterol. All data are presented as the mean ± s.d. (*n* = 3). Statistical significance was analyzed by one-way ANOVA. *P <* *0.05, **P <* *0.001, ***P <* *0.0001.

We further examined the *in vivo* distribution of the optimal mRNA (*FLuc*)-LNP formulation, N4 and the original Spikevax-type mRNA-LNP formulation, MS, before and after lyophilization in C57BL/6 mice, which were transfected intravenously (Figure 6A). Freshly prepared and reconstituted lyophilized mRNA-LNPs (0.2 µg mRNA (*FLuc*)/µl) of N4 and MS were administered via intravenous injection in C57BL/6 mice (*n* = 3). The mRNA (*FLuc*) entrapped in commercially available Lipofectamine 2000 (mRNA-Lipo) served as a positive control. Six hours later, d-luciferin potassium salt was administered intraperitoneally for luminescence visualization, followed by dissection and imaging of the mouse organs. The bioluminescence signals of the major organs (the heart, liver, spleen, lung, kidneys and eyes) were evaluated using an imaging system (IVIS) 6 hours after injection. The results are shown in Figure 6B–E.

The bioluminescence signals in the major organs of C57BL/6 mice treated by the N4, MS and mRNA-Lipo groups decreased from 2.5 × 10^5^‒1 × 10^6^ to 1 × 10^4^‒5 × 10^4^ after lyophilization (Figure 6D and E). The MS and N4 primarily transfected into the liver and spleen both before and after lyophilization, with no bioluminescence signals detected in the heart, kidneys or eyes. mRNA-Lipo was primarily transfected into the spleen both before and after lyophilization. Notably, fresh N4 exhibits significantly higher TE in the lungs compared to MS or mRNA-Lipo, resulting in notably higher bioluminescence signals in the lungs.

In particular, freshly prepared N4 presented a slightly greater bioluminescence signal in the spleen than MS did and a significantly greater bioluminescence intensity than mRNA-Lipo did (Figure 6B). Freshly prepared MS presented significantly greater bioluminescence signals in the liver, compared with those of mRNA-Lipo and N4. After lyophilization, reconstituted lyophilized N4 presented significantly greater bioluminescence signals in the spleen than MS and mRNA-Lipo did. These results indicated the optimized mRNA-LNPs N4 as a compelling candidate for the treatment of lung-specific diseases.

One limitation of this study is the reduced TE observed after lyophilization, likely due to structural changes during the drying process. Future work will aim to optimize lyophilization protocols to maintain formulation integrity. Another potential explanation is altered metabolism rates of freshly prepared versus reconstituted lyophilized mRNA-LNPs *in vivo*, which could affect bioluminescence signal intensity at the same detection time point. Further research should evaluate bioluminescence signal intensity at various time points to systematically investigate the *in vivo* metabolism of freshly prepared and reconstituted lyophilized mRNA-LNPs.

Another limitation of this study is that the *in vivo* translatability of lyophilized mRNA-LNPs after long-term storage has not been fully determined. The duration for which lyophilized mRNA-LNPs can maintain their *in vivo* translatable efficiency requires further investigation. However, based on the highly retained *in vitro* transfection efficiencies observed after long-term storage (0, 1, 4, 8 and 12 weeks) (Supplementary Figure S7), where the average TE of lyophilized mRNA-LNPs (N4) in HEK-293T cells remained at 98% after 12 weeks. Thus, we predict that lyophilized mRNA-LNPs can maintain their efficacy for at least 3 months. In addition, in our previous study [28], which optimized a Comirnaty-type lyophilized mRNA-LNP formulation, the optimal mRNA-LNP (sample O9) successfully induced fluorescent protein expression *in vivo* after 8 weeks of storage at 4°C, which is consistent with the *in vitro* TE experiments. However, organ tropism appeared to change over time. This suggests that while lyophilized mRNA-LNPs can retain translatability, their potential influence on *in vivo* biodistribution and metabolic stability should be further investigated.

Additionally, discrepancies were observed between *in vivo* results and transfection efficiencies in various tissue-derived cells *in vitro*. For instance, reconstituted lyophilized N4 demonstrated high TE in a lung-derived cell line (A549) *in vitro*, yet no bioluminescence signals were detected in the lungs after intravenous injection. This suggests that multiple biological barriers *in vivo* may impede the distribution of mRNA-LNPs [50, 51].

## Conclusions

We successfully developed an mRNA-LNP formulation (N4) by using the mathematical models established through I-optimal design of mixture experiments. The optimized formulation of N4 has significantly enhanced stability at 4°C after lyophilization by optimizing the four lipid components of the mRNA-LNPs. This formulation not only has high TE in HEK-293T cells, which surpasses that of the original Spikevax^@^ vaccine formulation, but also has high TE in the retinal ARPE-19 cell line, indicating its potential for treating hereditary ocular diseases. *In vivo* experiment revealed that the optimized formulation of N4 exhibits outstanding TE in the spleen in a mouse model after intravenous injection, with reduced liver expression, particularly in its lyophilized form. Notably, freshly prepared N4 generates notably higher bioluminescence signals in the lungs compared to MS or mRNA-Lipo. These findings suggest that N4 has potential for targeted gene delivery in therapeutic applications for pulmonary and spleen-specific disorders, paving the way for improved treatment strategies. The broad applicability of the optimized formulation of mRNA-LNPs across cell lines and tissues provides a new development direction and stability solution for LNP-based gene therapy, demonstrating its potential for application in future RNA delivery systems for gene therapy.

## Supplementary Material

rbaf023_Supplementary_Data

## Data Availability

All relevant data supporting the key findings of this study are available within the article and its Supplementary Information files or from the corresponding author upon reasonable request. Source data are provided with this paper.
