# Supplementary material for: Development of lyophilized mRNA-LNPs with high stability and transfection efficiency in specific cells and tissues
Source: Regen Biomater. 2025 Apr 10;12:rbaf023. doi: 10.1093/rb/rbaf023 (PMC12365600; doi:10.1093/rb/rbaf023)
Supplement: rbaf023_Supplementary_Data [file rbaf023_supplementary_data.docx]

**Development of Lyophilized mRNA-LNPs with High Stability and Transfection Efficiency in Specific Cells and Tissues**

Ting Wang^1, #^, Tao Yu^1, #^, Wanqi Li^1^, Jianyang Chen^1^, Sitian Cheng^1^, Zeyu Tian^1^, Tzu-Cheng Sung^1^, and Akon Higuchi^1-3,^ *

^1^State Key Laboratory of Ophthalmology, Optometry and Visual Science, Eye Hospital, Wenzhou Medical University, No. 270, Xueyuan Road, Wenzhou, Zhejiang, 325027, China

^2^Department of Chemical and Materials Engineering, National Central University, No. 300, Jhongda RD., Jhongli, Taoyuan 32001, Taiwan

^3^R&D Center for Membrane Technology, Chung Yuan Christian University, Chungli, Taoyuan 320, Taiwan

* Correspondence and requests for materials should be addressed to A.H. (e-mail: higuchi@ncu.edu.tw & higuchi@wmu.edu.cn)

Address: State Key Laboratory of Ophthalmology, Optometry and Visual Science, Eye Hospital, Wenzhou Medical University, No. 270, Xueyuan Road, Wenzhou, Zhejiang, 325027, China & Department of Chemical and Materials Engineering, National Central University, No. 300, Jhongda RD., Jhongli, Taoyuan 32001, Taiwan China.

^#^ These authors contributed equally to this work

Supplementary Information

**Supplementary Table S1.** Pre-set range of molar ratio of lipid components in LNPs

| Lipids | Range of molar ratio | Original lipid molar ratio in Spikevax^@^ vaccine |
| --- | --- | --- |
| SM-102 | 15~65% | 50 |
| Cholesterol | 15~65% | 38.5 |
| DSPC | 5~40% | 10 |
| DMG-PEG_2000_ | 1~10% | 1.5 |

**Supplementary Table S2.** Example of sequence detection model parameters

| Mixture Order | Model Parameter |
| --- | --- |
| Linear | $aA+bB+cC+dD$ |
| Quadratic | $aA+bB+cC+dD+eAB+fAC+gAD+hBD+iCD$ |
| Special Cubic | $aA+bB+cC+dD+eAB+fAC+gAD+hBD+iCD+jABC+kABD+lACD+mBCD$ |

**Supplementary Table S3.** Simplified model parameters

| Model | Model Parameter |
| --- | --- |
| Florescence intensity | $aA+bB+cC+dD+eAB+fAC$ |
| Encapsulation efficiency | $aA+bB+cC+dD$ |
| Diameter | $aA+bB+cC+dD+eAB+fAC+gAD+hBD+iCD$ |

**Supplementary Table S4.** Detailed information on the cell culture medium, centrifugation parameters and digestion time for each cell line used in this study

| Cell lines | Basal medium | Cat. # | Centrifugal parameter | Digestion time |
| --- | --- | --- | --- | --- |
| HEK-293T | Gibco™ DMEM, high glucose, GlutaMAX™ Supplement + 10% FBS | 10566016 | 1000 rpm, 4 min | 20 s |
| Colo205 | Gibco™ BASIC RPMI 1640 Medium + 10% FBS | 11875093 | 1000 rpm, 4 min | 2~3 min |
| PC12 | Gibco™ BASIC RPMI 1640 Medium + 10% FBS | 11875093 | 1000 rpm, 4 min | 2~3 min |
| Y79 | Gibco™ BASIC RPMI 1640 Medium + 10% FBS | 11875093 | 1000 rpm, 4 min | 30 s |
| ARPE19 | Dulbecco's Modified Eagle Medium/Nutrient Mixture F-12 (DMEM/F12) + 10% FBS | 10565018 | 1000 rpm, 5 min | 2~4 min |
| A549 | Gibco™ BASIC Ham's F-12 Nutrient Mix (F12) + 10% FBS | 11765054 | 1000 rpm, 5 min | 2~4 min |
| HepG2 | Gibco™ DMEM, high glucose, GlutaMAX™ Supplement + 10% FBS | 10566016 | 1000 rpm, 5 min | 2~4 min |

**Supplementary Table S5.** Validation of the accuracy of the encapsulation efficiency related model by comparing the actual encapsulation efficiency of the original Spikevax-type mRNA-LNPs with the predicted values

| Actual Value | Predicted Value | Residual | Externally Studentized Residuals |
| --- | --- | --- | --- |
| 66.71 | 76.11 | -9.4 | -2.63 |
| 82.04 | 76.11 | 5.93 | 1.66 |
| 86.01 | 76.11 | 9.9 | 2.77 |

**Supplementary Table** S**6.** Validation of the accuracy of the diameter related model by comparing the actual diameter of the original Spikevax-type mRNA-LNPs with the predicted values

| Actual Value | Predicted Value | Residual | Externally Studentized Residuals |
| --- | --- | --- | --- |
| 115.8 | 108.74 | 7.06 | 0.7055 |
| 118.1 | 108.74 | 9.36 | 0.9355 |
| 124.2 | 108.74 | 15.46 | 1.55 |

**Supplementary Table S7.** Validation of the accuracy of the fluorescence intensity related model by comparing the actual normalized median fluorescence intensity (nMFI) values of the original Spikevax-type mRNA-LNPs with the predicted values.

| Actual Value | Predicted Value | Residual | Externally Studentized Residuals |
| --- | --- | --- | --- |
| 0.4666 | 0.4077 | 0.0589 | 1.74 |
| 0.2851 | 0.4077 | -0.1226 | -3.61 |
| 0.2538 | 0.4077 | -0.1539 | -4.54 |

**Supplementary Table S8.** The optimized parameters of mathematic modeling

| Name | Goal | Lower Limit | Upper Limit | Lower Weight | Upper Weight | Importance |
| --- | --- | --- | --- | --- | --- | --- |
| A:DSPC | is in range | 0.05 | 0.4 | 1 | 1 | 3 |
| B:SM-102 | is in range | 0.15 | 0.65 | 1 | 1 | 3 |
| C:Cholesterol | is in range | 0.15 | 0.65 | 1 | 1 | 3 |
| D:DMG-PEG_2000_ | is in range | 0.01 | 0.1 | 1 | 1 | 3 |
| EE | maximize | 10.63 | 98.46 | 1 | 5 | 4 |
| nMFI | minimize | 0.86 | 3.48 | 1 | 1 | 5 |

Note: EE represents encapsulation efficiency; nMFI represents normalized median fluorescence intensity.

**Supplementary Table S9.** The predicted candidates of mRNA-LNPs with pre-set lipid molar ratio and encapsulation efficiency as well as nMFI by the established models

| Number | DSPC | SM-102 | Cholesterol | DMG-PEG_2000_ | EE | nMFI | Desirability |
| --- | --- | --- | --- | --- | --- | --- | --- |
| **C1** | **0.29** | **0.65** | **0.05** | **0.01** | **83.23** | **4.298** | **0.821** |
| C2 | 0.19 | 0.15 | 0.65 | 0.01 | 95.73 | 1.442 | 0.695 |
| C3 | 0.05 | 0.47 | 0.47 | 0.01 | 84.20 | 2.068 | 0.595 |
| C4 | 0.314 | 0.15 | 0.526 | 0.01 | 86.35 | 1.436 | 0.534 |

Note: EE represents encapsulation efficiency; nMFI represents normalized median fluorescence intensity.

**Supplementary Table S10.** Detailed composition ratio information for mRNA-LNPs with various molar ratios of DSPC and cholesterol

| Sample No. | DSPC | SM-102 | Cholesterol | DMG-PEG_2000_ | | DSPC/Cholesterol |
| --- | --- | --- | --- | --- | --- | --- |
| N1 (C1) | 0.29 | 0.65 | 0.05 | | 0.01 | 5.8 |
| N2 | 0.24 | 0.65 | 0.10 | | 0.01 | 2.4 |
| N3 | 0.19 | 0.65 | 0.15 | | 0.01 | 1.27 |
| N4 | 0.09 | 0.65 | 0.25 | | 0.01 | 0.36 |
| N5 | 0.04 | 0.65 | 0.30 | | 0.01 | 0.13 |

**Supplementary Table S11.** The average zeta potential of mRNA encapsulated in different vectors before and after lyophilization.

| **Parameters** | **Sample format** | **mRNA encapsulated in different vectors** | | | |
| --- | --- | --- | --- | --- | --- |
|  |  | mRNA + Lipofectamine 2000 | MS | N1 | N4 |
| **Zeta potential (mV) ± STD** | **fresh** | -12.5 ± 0.45 | 5.8 ± 0.17 | 8.0 ± 0.28 | 7.6 ± 0.40 |
|  | **lyophilized** | -17.4 ± 1.21 | 8.2 ± 0.08 | 4.3 ± 0.77 | 9.6 ± 0.74 |





**Supplementary Figure S1.** PDI value of each designed mRNA-LNP and MS.


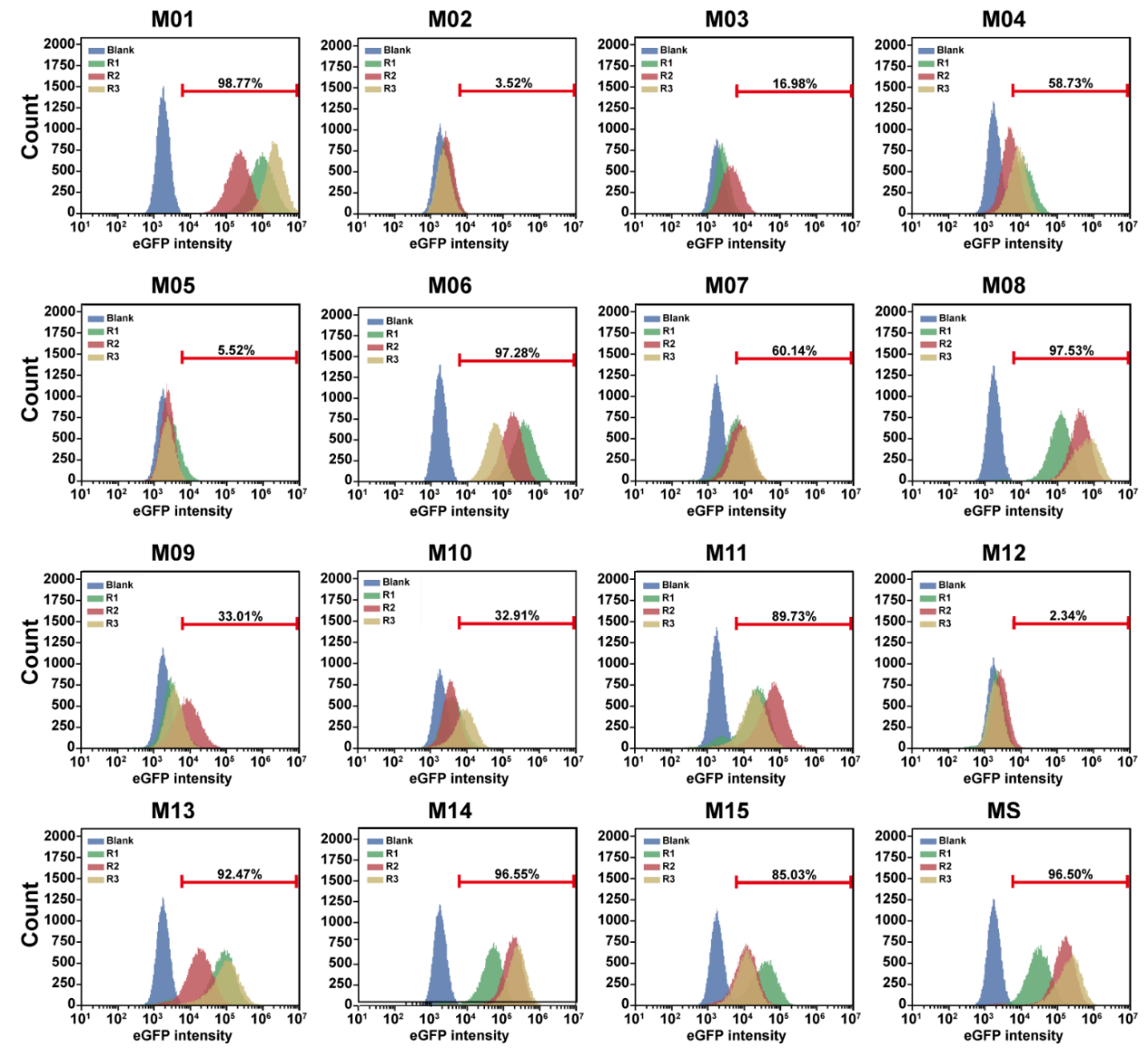


**Supplementary Figure S2.** Representative flow cytometry plots for the expression of eGFP on HEK-293T cells transfected with freshly prepared (*eGFP*) mRNA-LNPs of the S type, which were designed by an I-optimal design. Each sample was evaluated by three parallel experiments (n = 3; R1, R2, and R3). The labeled transfection efficiencies are the average transfection efficiency of three independent assays.


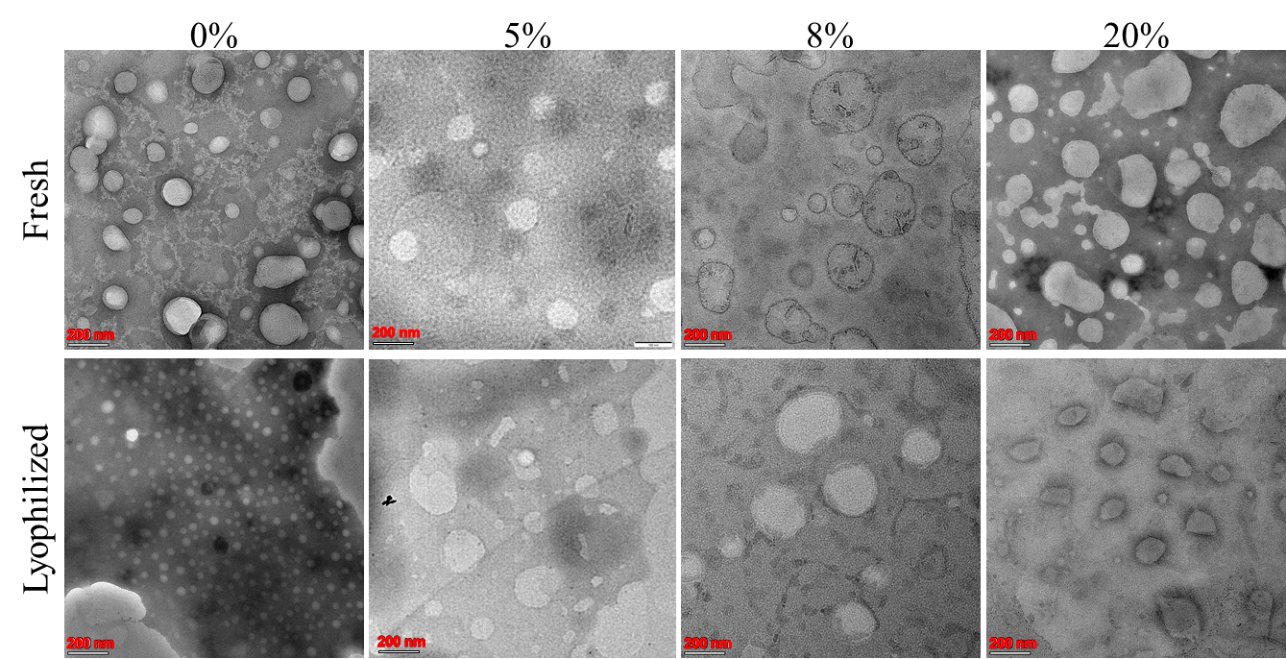


**Supplementary Figure S3.** Representative TEM images showing the effects of sucrose concentration (0%, 5%, 8%, and 20%) on the structural integrity and morphology of (*eGFP*) mRNA-LNPs (M01) before and after lyophilization. The scale bar represents 200 nm.


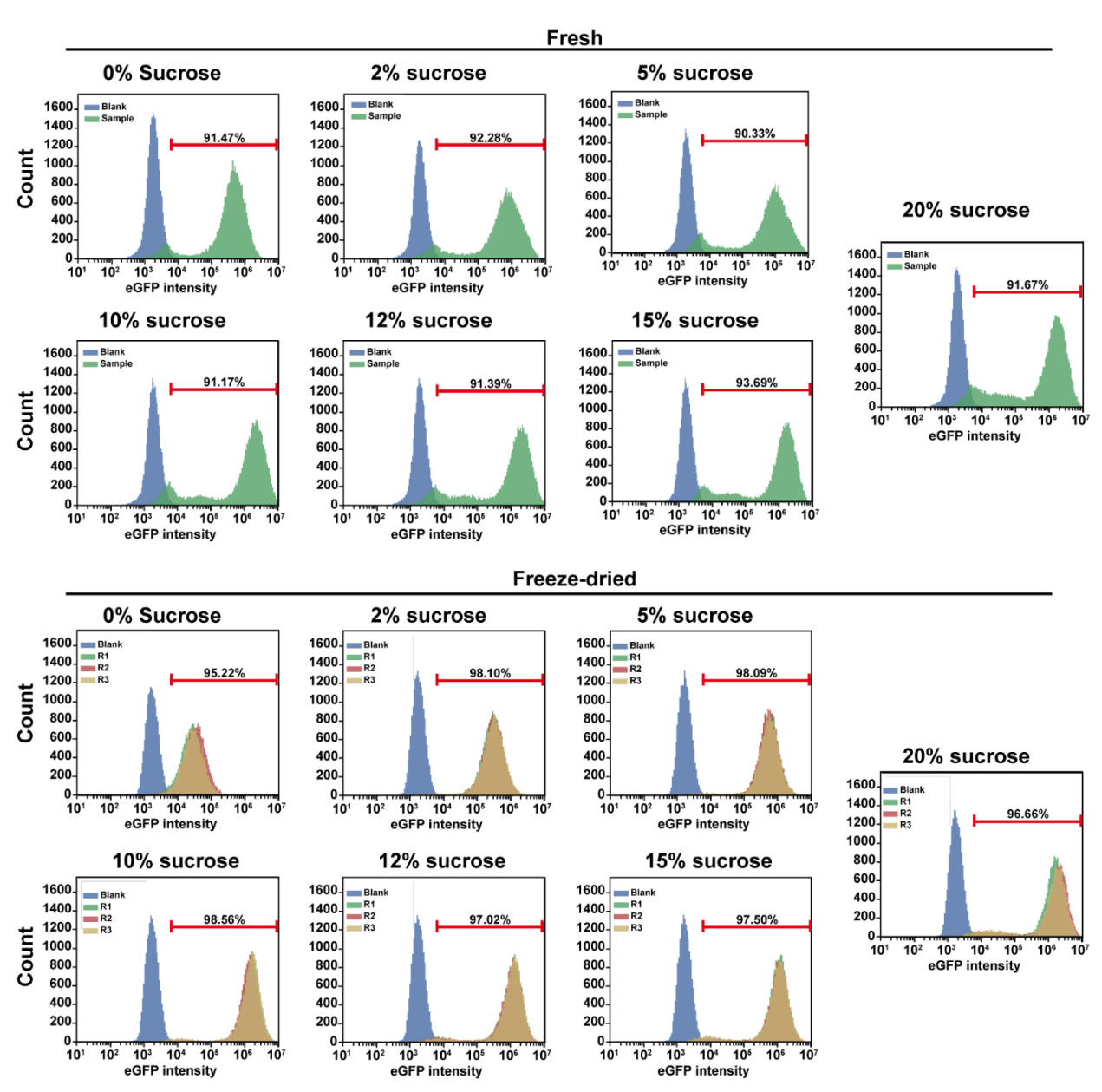
**Supplementary Figure S4.** Representative flow cytometry plots for the expression of eGFP on HEK-293T cells transfected with freshly prepared (fresh) mRNA(*eGFP*)-LNPs and lyophilized (freeze-dried) mRNA(*eGFP*)-LNPs after reconstitution, which contained various concentrations (0%, 2%, 5%, 10%, 12%, 15%, 20%) of sucrose. Each sample was evaluated by three parallel experiments (R1, R2, and R3). The labeled transfection efficiencies were the average transfection efficiency of three independent assays.


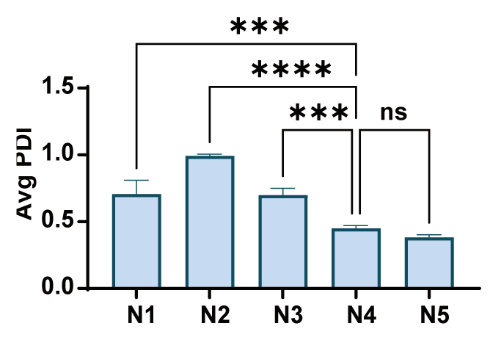


**Supplementary Figure S5.** The average PDI value of reconstituted lyophilized mRNA-LNPs with various DSPC/cholesterol ratios (5.8, 2.4, 1.27, 0.36, and 0.13) after storage for 0, 1, 4, 8, and 12 weeks.


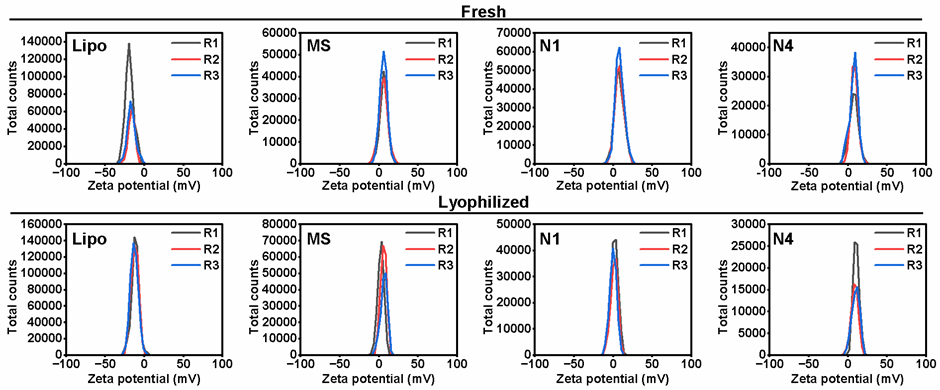


**Supplementary Figure S6.** Comparisons of zeta potential of representative mRNA-LNP (N1), optimal mRNA-LNP (N4), original mRNA-LNP (MS), and Lipofectamine 2000-encapsulated (*eGFP*) mRNAs (Lipo) before and after lyophilization. Three repeated measurements (R1, R2, R3) were performed.


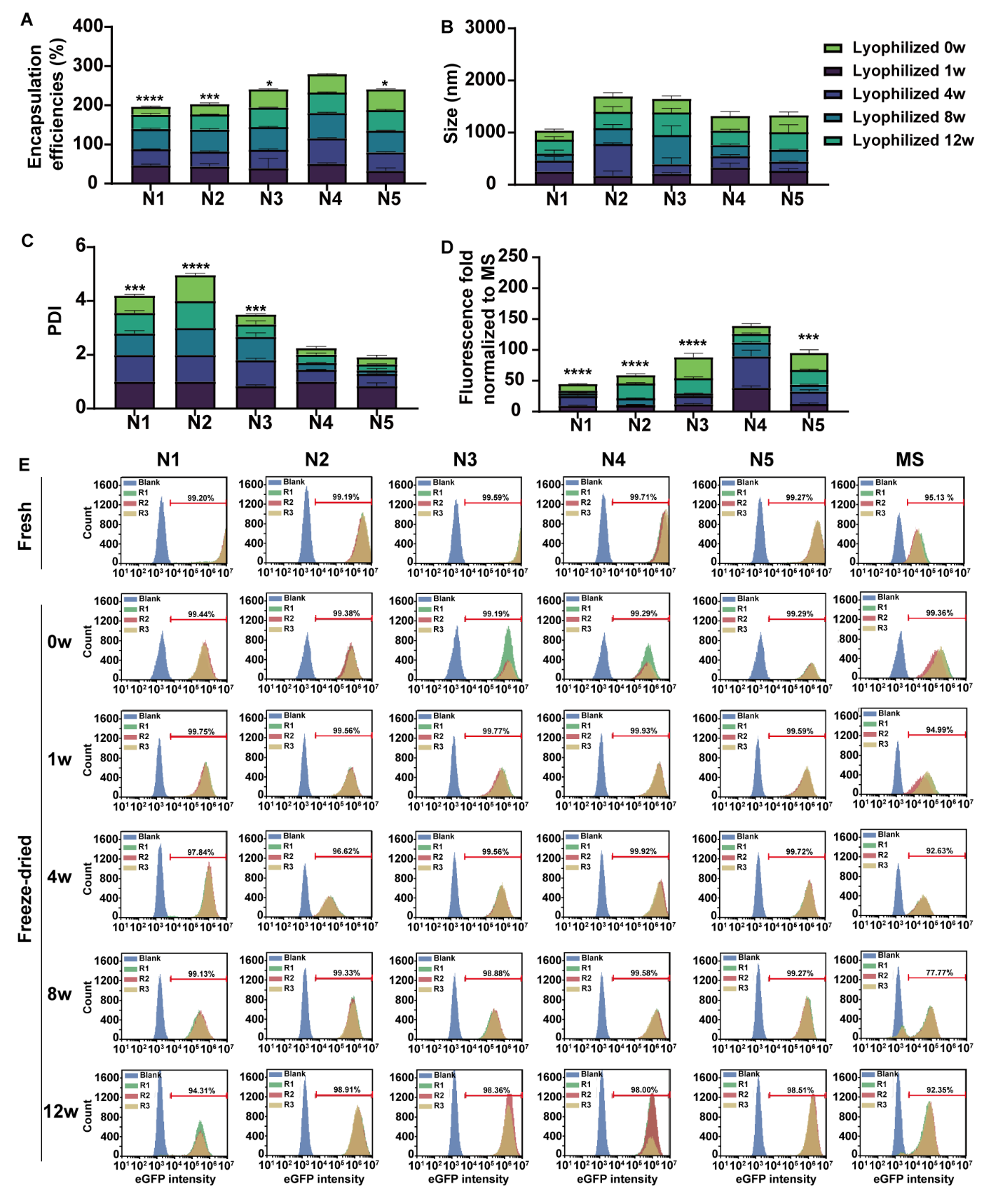


**Supplementary Figure S7.** Evaluation of the influence of the DSPC-to-cholesterol ratio (DSPC/Cho) on the long-term storage stability of mRNA (*eGFP*)-LNPs at 4 °C after lyophilization. (A) The accumulated encapsulation efficiency of reconstituted lyophilized mRNA-LNPs with various DSPC/Cho ratios (5.8, 2.4, 1.27, 0.36, and 0.13) after storage for 0, 1, 4, 8, and 12 weeks. (B, C) The accumulated particle size (B) and PDI value (C) of reconstituted lyophilized mRNA (*eGFP*)-LNPs with various DSPC/Cho ratios (5.8, 2.4, 1.27, 0.36, and 0.13) after storage for 0, 1, 4, 8, and 12 weeks. (D) The accumulated fluorescence fold normalized to MS in HEK-293T cells treated with reconstituted lyophilized mRNA-LNPs with different DSPC/Cho ratios after storage for 0, 1, 4, 8, and 12 weeks, as evaluated by flow cytometry. (E) Representative flow cytometry plots for the expression of eGFP on HEK-293T cells transfected with fresh and reconstituted lyophilized mRNA (*eGFP*)-LNPs with various DSPC/Cho ratios (5.8, 2.4, 1.27, 0.36, and 0.13) after storage for 0, 1, 4, 8, and 12 weeks. Each sample was evaluated by three parallel experiments (R1, R2, and R3). All the data are presented as the mean ± s.d. (n = 3). Statistical significance was analyzed by one-way ANOVA. *p<0.05; ***p<0.0005; ****p<0.0001.


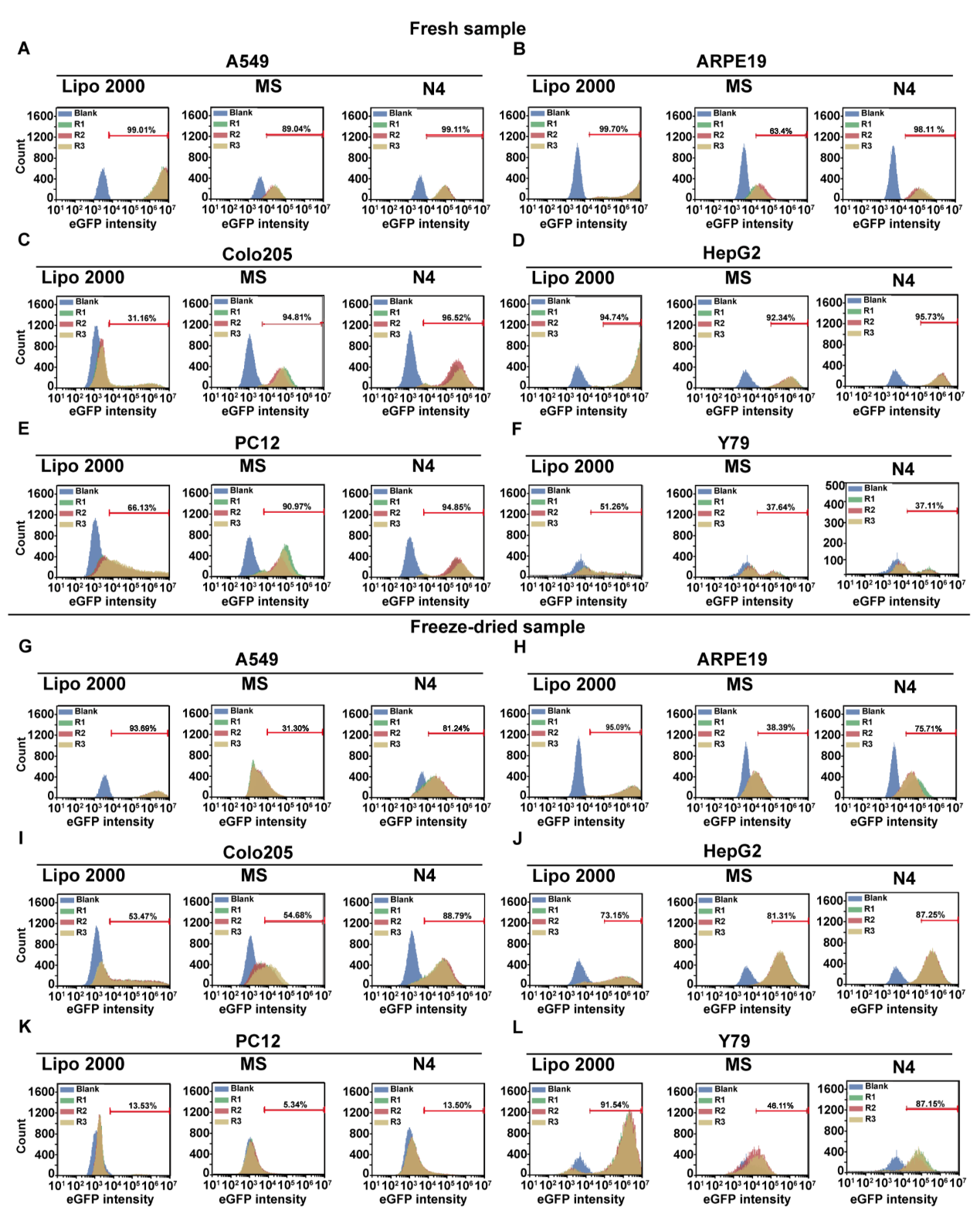


**Supplementary Figure S8.** Comparisons of transfection efficiency for optimized mRNA-LNP (N4), original mRNA-LNP (MS), and Lipofectamine 2000-encapsulated (*eGFP*) mRNAs (labeled as Lipo 2000) in various cell lines derived from different organs. Representative flow cytometry plots for the expression of eGFP in various cell lines derived from different organs (A and G, A549 cells; B and H, ARPE19 cells; C and I, Colo205 cells; D and J, HepG2 cells; E and K, PC12 cells; F and L, Y79 cells) , which is transfected with fresh (A-F) and reconstituted lyophilized (G-L) optimized mRNA-LNP (N4), original mRNA-LNP (MS), and Lipofectamine 2000-encapsulated (*eGFP*) mRNAs. Each sample was evaluated by three parallel experiments (R1, R2, and R3). The labeled transfection efficiencies are the average transfection efficiency of three independent assays.





**Supplementary Figure S9.** Cell viability of the tested cells transfected with fresh prepared (A) or reconstituted lyophilized (B) mRNA-LNP of N4 and MS, as well as lipo-mRNA was detected by CCK-8 assays.
